# A Meta-Analysis of Predation Risk Effects on Pollinator Behaviour

**DOI:** 10.1371/journal.pone.0020689

**Published:** 2011-06-13

**Authors:** Gustavo Q. Romero, Pablo A. P. Antiqueira, Julia Koricheva

**Affiliations:** 1 Departamento de Biologia Animal, Instituto de Biologia (IB), Universidade Estadual de Campinas (UNICAMP), Campinas, Brazil; 2 Pós-graduação em Biologia Animal, IBILCE, Universidade Estadual Paulista (UNESP), São José do Rio Preto, Brazil; 3 School of Biological Sciences, Royal Holloway, University of London, Egham, United Kingdom; Institut Mediterrani d'Estudis Avançats (CSIC/UIB), Spain

## Abstract

Flower-visiting animals are constantly under predation risk when foraging and hence might be expected to evolve behavioural adaptations to avoid predators. We reviewed the available published and unpublished data to assess the overall effects of predators on pollinator behaviour and to examine sources of variation in these effects. The results of our meta-analysis showed that predation risk significantly decreased flower visitation rates (by 36%) and time spent on flowers (by 51%) by pollinators. The strength of the predator effects depended neither on predator taxa and foraging mode (sit-and-wait or active hunters) nor on pollinator lifestyle (social vs. solitary). However, predator effects differed among pollinator taxa: predator presence reduced flower visitation rates and time spent on flowers by Squamata, Lepidoptera and Hymenoptera, but not by Diptera. Furthermore, larger pollinators showed weaker responses to predation risk, probably because they are more difficult to capture. Presence of live crab spiders on flowers had weaker effects on pollinator behaviour than presence of dead or artificial crab spiders or other objects (e.g. dead bees, spheres), suggesting that predator crypsis may be effective to some extent. These results add to a growing consensus on the importance of considering both predator and pollinator characteristics from a community perspective.

## Introduction

Predation has long been considered as one of the central topics in ecology. A classic view is that predators cause changes in population, community and food web attributes via consumptive effects (i.e., the density mediated interaction concept). Non-consumptive effects of predation, defined as changes in prey traits under predation risk (i.e., the trait mediated interaction concept), have received considerable attention only in recent years [1]–[3]. It has been shown that the magnitude of non-consumptive effects can be similar to or even higher than that of consumptive effects [3]. When the distribution of resources is predictable but distribution of predators is not, foraging animals face a trade-off between acquiring appropriate food and avoiding predation [4]. Given that costs of errors in detecting predation risk are very high [5], even low predation levels may impose a strong selection pressure on pollinators to develop anti-predatory adaptations [6], [7].

Although predator avoidance behaviour by flower visitors has been described already 50 years ago [8], active research on this topic began only in the last few years [9]–[16]. It has been shown that changes in pollinator behaviour can be triggered by various types of predators, such as crab spiders [10], [17], [18], phymatid ambush bugs [19], ants [20], wasps [13], dragonflies [16] or vertebrates [e.g., 21] and that effects of predators on pollinator behaviour range from negligible [22] to strong [16]. Moreover, pollinators can recognize parts of predator body (e.g., crab spider forelimbs; [23]), and detect past predation events on flowers [9]. Furthermore, pollinators can learn and memorize information on dangerous flowers [14], [15], and even transmit information on predation risk to conspecifics [9]. In addition, some predators can be cryptic and often use behavioural tactics and physiological machinery to minimise detection (e.g., [24], but see [11]) or even manipulate flower signals to lure pollinators [25]. However, to date few studies have examined the extent to which pollinator behaviour depends on predator and pollinator traits [29].

We conducted a meta-analysis of published and unpublished data on effects of predation risk on pollinator behaviour in order to estimate overall effects as well as to identify potential sources of variation in pollinator responses. Our first prediction concerns predator foraging mode; we predict that cues from stationary predators (sit-and-wait) could be more indicative of imminent predation risk and thus should trigger stronger behavioural responses from pollinators than cues from actively hunting predators [26], [27]. We also predict that the magnitude of the effect on pollinator behaviour will be lower in the case of live predators, which can match background colours of flowers and display cryptic behaviours (e.g., crab spiders, [11]), as compared to the effect of presence of the dead or artificial predators, which lack crypsis. Given the independent evolutionary histories and the differences in each group's lifestyles across pollinator and flower-dwelling predator taxa, we also expect variable abilities to detect predation risk among pollinators and variable effects on pollinator avoidance behaviours among predators. For instance, pollinator taxa with lower visual acuity (e.g., beetles) may be less likely to display predator avoidance behaviour than pollinators with more highly developed vision (e.g., bees, flies). Since small pollinators are more likely to be captured than larger ones (reviewed by [28]), we expect larger behavioural changes in response to predation risk in smaller pollinators. In addition, we predict that social Hymenoptera (e.g., honeybees) display higher accuracy in locating dangerous flowers than the solitary ones, since by experiencing predation risk they can steer naïve individuals in the colony to recruit away from dangerous flowers [9].

## Methods

### Data collection, inclusion criteria and sources of variation

We searched the literature reporting effects of predators on floral visitor behaviour using the expanded database from Science Citation Index (isiknowledge.com). We used the following keywords “pollin* and predat*”, “predation risk and pollinat*”, “pollinat* and behav* and predat*”, “pollin* and risk sensit*”, “avoidance behav* and pollin*”, “phymat* and pollin*”, “manti* and pollin*”, “thomisid* and pollin*”, “ant* and pollin* and behav*”, “wasp* and pollinat* and behav*”, “dragonfl* and pollin*”, “bird* and pollin* and behav*”, and “lizard* and pollin* and behav*”. We have also done haphazard searches in Google Scholar using the keywords listed above. In addition, we examined reference lists of the papers found, as well as studies included in recent literature reviews on the topic [12], [27]–[32]. Unpublished data from several experiments conducted in neotropical forests in Brazil were also included in our meta-analysis; detailed material and methods describing the systems and experimental designs for the unpublished data is presented in Appendix S1. Although information provided in many studies was insufficient to discriminate whether flower visitors are true pollinators in the system, for the purpose of this study we took a pragmatic approach and considered all flower visitors as potential pollinators; hence we use the term “pollinator” to refer to any floral visitor thereafter.

We considered both experimental and observational studies that have provided a comparison of flower visitation rate (i.e., number of visits per unit time), or time spent foraging on flowers or inflorescences (response variables) on plants with and without predators. Experimental studies directly manipulated predator presence or absence on flowers (by introducing or removing predators), whereas observational studies compared pollinator behaviour on sites or flowers where carnivores were naturally present or absent. Plants with predators present could have one or several predators, but we could not include predator density in the analysis because many studies have not reported predator numbers per flower. We only included studies that compared plants with and without predators simultaneously. Some studies reported the effect of presence of structures other than live predators (e.g., dead or artificial predators, dead pollinators or any other abiotic object on flowers) on pollinator behaviours; these cases were included in the analyses for comparisons. Data on density or abundance of flower visitors (e.g., number of insects per flower) were not included in the analysis because they do not provide accurate responses on predation risk. The list of papers that matched the above inclusion criteria is presented in the Appendix S2. We also included unpublished data on flower visitation rate and avoidance rate of pollinators (see Appendix S1 for methods on data samplings); avoidance was defined as the situation when the insect approaches the flower by flying but instead of landing on it switches to another flower or leaves the area (see [23] and Appendix S1).

The sources of variation evaluated were predator hunting mode, predator taxa, predation risk, pollinator taxa (Order), pollinator lifestyle (solitary vs. social), and pollinator size (biomass). Predator taxa compared were ants, birds, crab spiders, dragonflies, lizards, ambush bugs (Phymatidae) and wasps. Predator hunting mode was classified as either sit-and-wait (i.e., crab spiders, ambush bugs, lizards) or active hunters (i.e., ants, dragonflies, wasps, birds). Predation risk categories included live, dead and artificial predators (models made using epoxy resin: [23] or paper: [33], and past predation event (PPE), i.e., presence of dead insects on flowers mimicking prey carcasses left by predators. We also included in the comparison effects of presence of any abiotic object on flowers (e.g., epoxy spheres; Appendix S1) to test the effects of structures that do not resemble predators. The effects of the above predation risk categories were examined only in studies on crab spiders, and therefore this analysis was restricted to crab spiders only. Pollinator orders compared were Hymenoptera (wasps, bees), Diptera, Lepidoptera, Coleoptera, Trochiliformes, Squamata and “several” when two or more orders were analyzed. Effects of pollinator lifestyle (solitary vs. social) were evaluated only for Hymenoptera because other pollinator taxa do not display social organization. Estimates on the interactions between ants (as predators) and geckos (Squamata) (as pollinators) differed from the rest of studies in this meta-analysis because ants are unlikely to prey on geckos and hence predation risk is very low. Yet, geckos display very strong avoidance behaviour in response to presence of ants on flowers. Full information on all studies included in the analysis, sources of variation, as well as effect sizes and associated variances are given in the supporting information (Tables S1, S2, S3, S4). Analysis of other sources of variation (native or invasive predators, pollinator family, experimental vs. observational studies) is presented in Appendix S3.

The final database consisted of 106 estimates of predator effects on pollinator visitation rates (62 estimates from 23 published papers plus 44 estimates from unpublished studies) and 37 estimates from 12 published studies on predator effects on time spent on flowers by pollinators. In addition, we analysed 32 unpublished estimates for flower avoidance rate by pollinators in the presence of predators (Appendix S1, S2, Table S2).

### Data extraction and meta-analysis

Many studies reported more than one estimate of effects of predators on pollinator behaviour, e.g. effects on visitation rates and time spent on flowers by different species of pollinators. These multiple estimates of the effect from the same study are statistically non-independent, but on the other hand we were interested in comparing predator effects on different pollinator taxa. As a compromise, if a study reported data on several species of pollinators, we have calculated the mean effect for all pollinator species belonging to the same family and included those family-specific values in the analysis. We considered family-level data from a single paper to be more independent, since large taxa evolve different abilities to evaluate predation risk on flowers (e.g., [11]). Some studies evaluated pollination behaviour under predation risk in different geographic regions or on different plant species. These data were treated as independent comparisons because (i) predators under study migrate relatively short distances and have short life cycles (locally restricted), and (ii) flowers of different plant species have variable colours, and predators can display variable degrees of crypsis depending on the flower colour background (e.g., [11]). To test whether larger pollinators are less responsive to predation risk, we used data on biomass of individual pollinator species from the literature and unpublished data (see Table S4). We categorized pollinator size as small (up to 50 g), medium (50.01 to 100 g) and large (>100 g) using fresh biomass data, since most of the data were on fresh biomass (75%, particularly from unpublished data). To estimate fresh mass from data on dry mass we collected 33 pollinator species belonging to several families and orders (Diptera, Hymenoptera) and estimated their fresh and dry mass. The resulting regression model (y = 4.15×−0.002, r^2^ = 0.99, P<0.001) allowed us to estimate fresh mass from pollinating insects of published studies.

Log response ratio (ln R) [34], [35] was used as a measure of the effect size. We then back-transformed ln R to % difference between control and treatment (as (EXP ln R −1)×100%) for the ease of interpretation. We also conducted analyses using standardized difference between the means, Hedges'd, as a metric of the effect size [36] to ensure that the results of our meta-analysis are not biased due to the choice of ln R as a metric. Since the results of the analysis were similar for both metrics, we report only ln R and corresponding % changes. We used mixed effect models because their assumptions are more likely to be satisfied in ecological data than those of fixed effect models [35]. Mixed effect models assume that there is a random variation among studies within a group, but variation among groups is fixed [35]. P values for the between-group heterogeneity (Q_b_) tests were obtained by randomization tests based on 4999 iterations. Confidence intervals (95%) were obtained by bootstrapping, and the effect sizes were considered significant if the CI did not overlap with the zero. The meta-analysis was carried out by using the MetaWin 2.0 statistical software [37]. Analyses of publication bias are presented in the Appendix S4.

## Results

Overall, the presence of live predators on flowers decreased pollinator visitation rate by 36% (ln R = −0.44, 95% CI = −0.63 to -0.27, n = 47) and the time spent by pollinators on flowers by 51% (mean ln R = −0.71, 95% CI = −1.11 to −0.39, n = 30).

**Figure 1:**
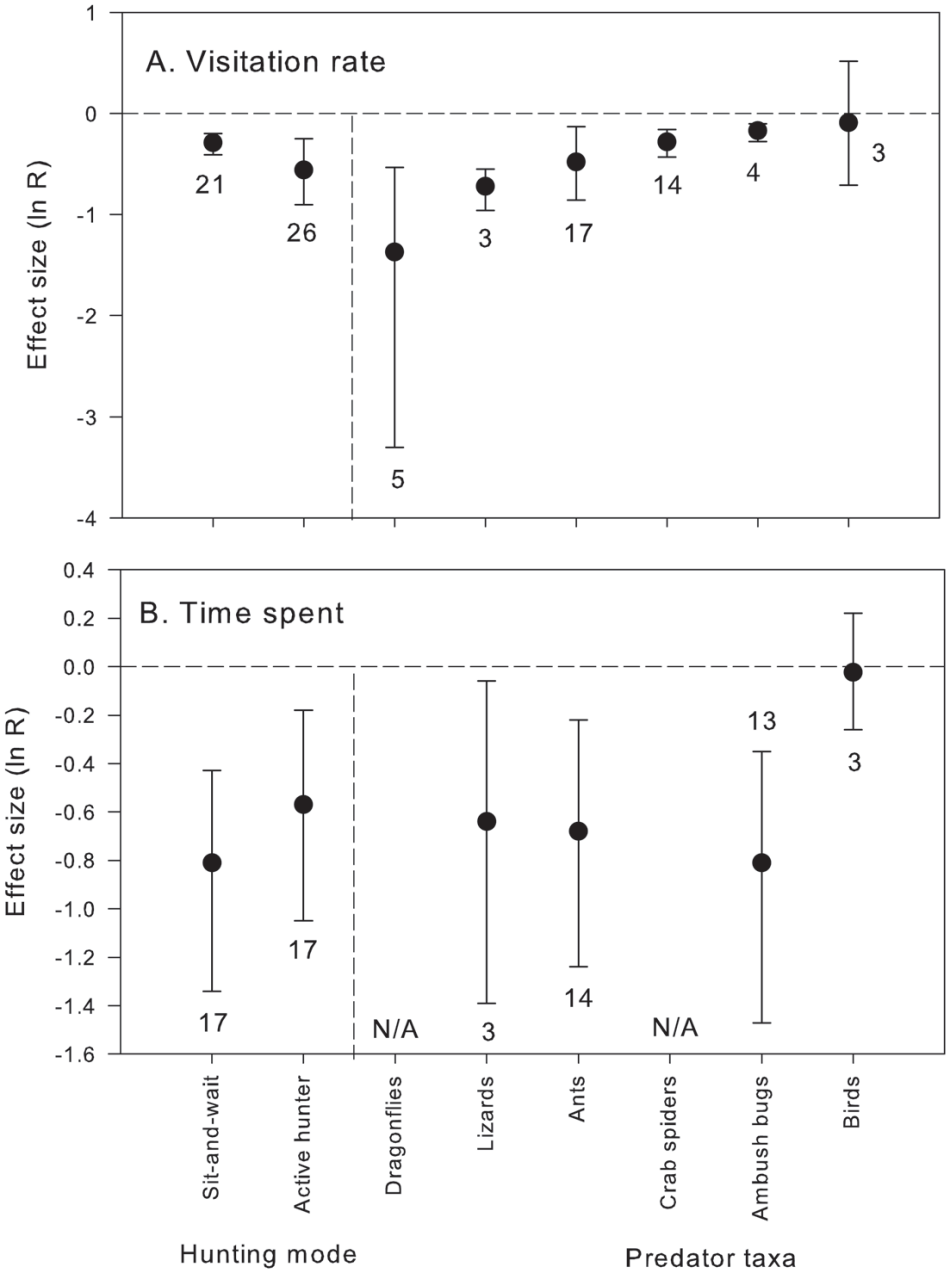
Effects (mean ln R and 95% CI) of predator hunting mode and taxa on (a) pollinator visitation rate and (b) time spent on flowers. Only live predators were analysed. Sample sizes are indicated next to the error bars. N/A = data not available. Negative effects indicate decrease in visitation rate or time spent on flowers with predators present; effects are considered significant if 95% CI does not include 0.

When only live predators were considered, predator hunting mode had no significant effect on pollinator behaviour (visitation rate: Qb = 2.2, P = 0.16, df = 1; time spent: Qb = 0.46, P = 0.480, df = 1). Both sit-and-wait predators and active hunters had strong negative effect on visitation rate of pollinators (Fig. 1a) and time the pollinators spent on flowers (Fig. 1b), indicating that both type of predators are similarly avoided by pollinators. Similarly, there were no significant differences among predator taxa in their effects on pollinator behaviour (visitation rate: Qb = 9.79, P = 0.136, df = 5; time spent: Qb = 1.27, P = 0.69, df = 3); all predator taxa except birds had a significant effect on pollinator behaviour (Fig. 1a, b).

**Figure 2:**
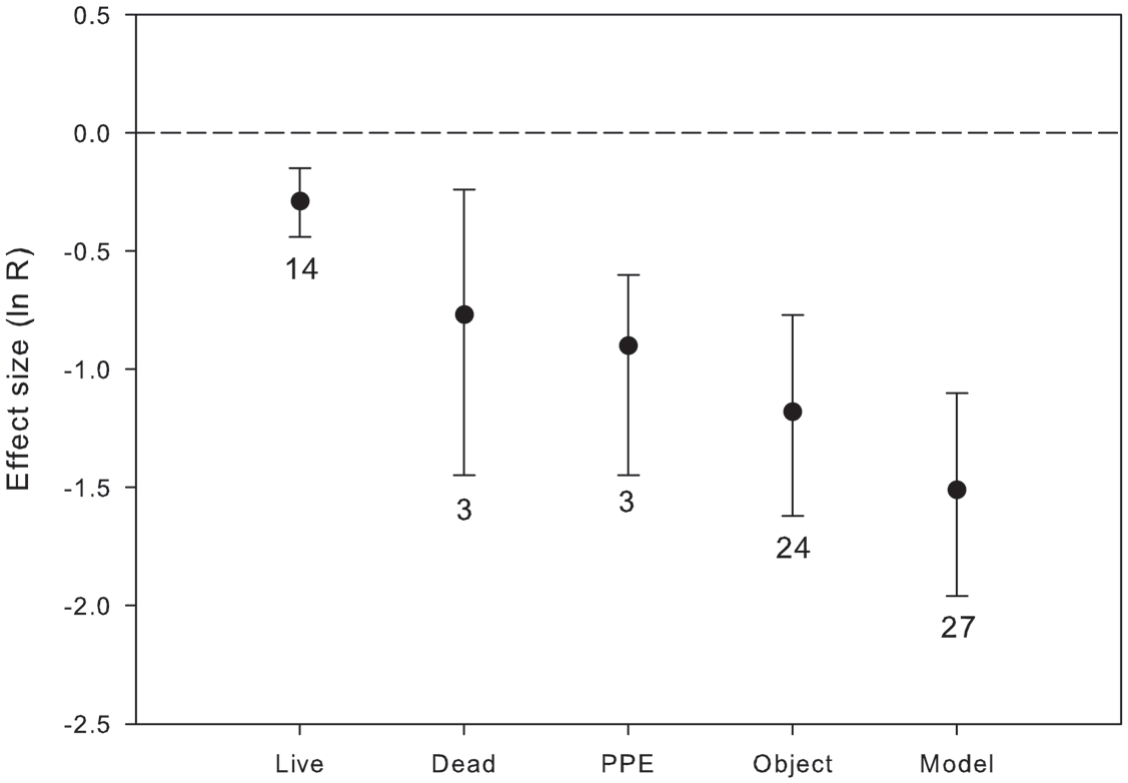
Effects (mean ln R and 95% CI) of live, dead, artificial crab spiders (model), past predation events (PPE) and objects that do no resemble predators on pollinator visitation rate of flowers. Sample sizes are indicated next to the error bars. Negative effects indicate decrease in visitation rate of flowers with predators present; effects are considered significant if 95% CI does not include 0.

We have compared the effects of live vs. dead crab spiders, artificial crab spider models, abiotic objects and past predation events (PPE, i.e. presence of dead insects on flowers mimicking prey carcasses typically left by crab spiders) (Fig. 2). There was high heterogeneity among the above categories for visitation rate (Qb = 22.85, P = 0.001, df = 4). Live crab spiders had the weakest effect on pollinators, decreasing visitation rate by 25%, while dead crab spiders and PPE decreased visitation rate by 54% and 59%, respectively. Even stronger effects were observed for abiotic objects on flowers (e.g., epoxy spheres) and artificial spiders: the former decreased visitation rate by 69% and the later by 78%. For avoidance rate, only data on artificial spiders and objects were available. Artificial spiders and objects did not differ from each other in their effects on visitation rate (Fig. 2), but differed in their effects on avoidance rate of pollinators (Qb = 4.77, P = 0.037, df = 1). While the presence of object on flowers increased avoidance rate by 209%, the presence of artificial spiders increased avoidance rate by 520% (artificial spider: ln R = 1.83, 95% CI = 1.53 to 2.22, n = 16; object: ln R = 1.13, 95% CI = 0.64 to 1.62, n = 16).

**Figure 3:**
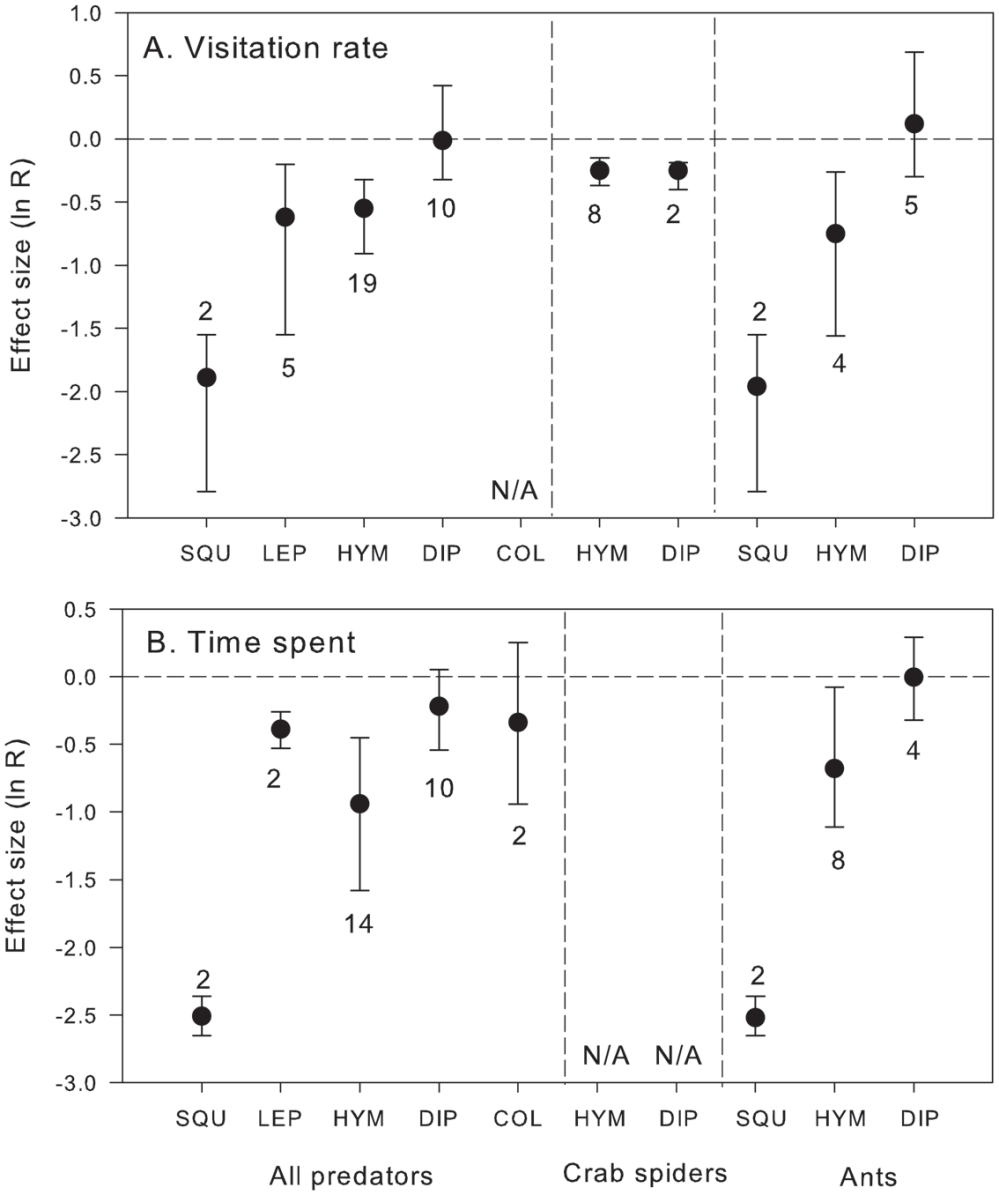
Effects (mean ln R and 95% CI) of all live predators, live crab spiders and ants on (a) visitation rate and (b) time spent on flowers by several pollinator taxa. Sample sizes are indicated next to the error bars. SQU = Squamata, LEP = Lepidoptera, HYM = Hymenoptera, DIP = Diptera, COL = Coleoptera. N/A = data not available. Negative effects indicate decrease in visitation rate or time spent on flowers with predators present; effects are considered significant if 95% CI does not include 0.

Effects of live predators on pollinator visitation rates differed significantly depending on pollinator order (Qb = 13.52, P = 0.020, df = 3). Visitation rates of geckonid lizards (Squamata) were affected most (85% decrease), followed by Lepidoptera (46% decrease) and Hymenoptera (42% decrease). In contrast, visitation rates by Diptera were not significantly affected (Fig. 3a). Effects of live predators on the time spent on flowers did not differ among pollinator orders (Qb = 9.32, P = 0.122, df = 4) although only Squamata, Lepidoptera, and Hymenoptera showed significantly reduced time spent on flowers in the presence of live predators, whereas Diptera and Coleoptera showed no significant response (Fig. 3b). Since pollinator order was not independent from predator taxa (e.g., all studies on Squamata examined effects of ants only), we ran separate analyses for the two most studied groups of predators, ants and crab spiders. While presence of crab spiders significantly decreased visitation rate by both Diptera and Hymenoptera (Qb<0.001, P = 0.98, df = 1), ants affected visitation rate and time spent on flowers by Hymenoptera, but not by Diptera (Fig. 3a,b; visitation rate: Qb = 9.62, P = 0.041, df = 2; time spent: Qb = 24.2, P = 0.002, df = 2).

Lifestyles of hymenopteran pollinators did not significantly affect their responses to predation risk (visitation rate: Qb = 1.30, P = 0.30, df = 1; time spent: Qb = 0.61, P = 0.37, df = 1). Both social and solitary Hymenoptera had lower visitation rates (social: ln R = −0.43, 95% CI = −0.84 to −0.19, n = 11; solitary: ln R = −0.79, 95% CI = −1.65 to −0.37, n = 8) and spent less time on flowers (social: ln R = −1.15, 95% CI = −2.10 to −0.53, n = 8; solitary: ln R = −0.56, 95% CI = −1.19 to 0.03, n = 6) in the presence of live predators, indicating that both lifestyles were affected in the presence of live predators. When we ran analyses only for social and solitary Apoidea (Apidae, Megachilidae, Colletidae, Halictidae, Andrenidae), the results were similar (visitation rate: Qb = 0.69, P = 0.44, df = 1; time spent: Qb = 0.011, P = 0.93, df = 1), i.e. both social and solitary bees visited fewer flowers ((social: ln R = −0.48, 95% CI = −0.95 to −0.22, n = 9; solitary: ln R = −0.77, 95% CI = −1.82 to 0.32, n = 7)) and spent less time on flowers in the presence of predators (social: ln R = −0.87, 95% CI = −1.23 to −0.42, n = 6; solitary: ln R = −0.83, 95% CI = −1.52 to −0.06, n = 4).

**Figure 4:**
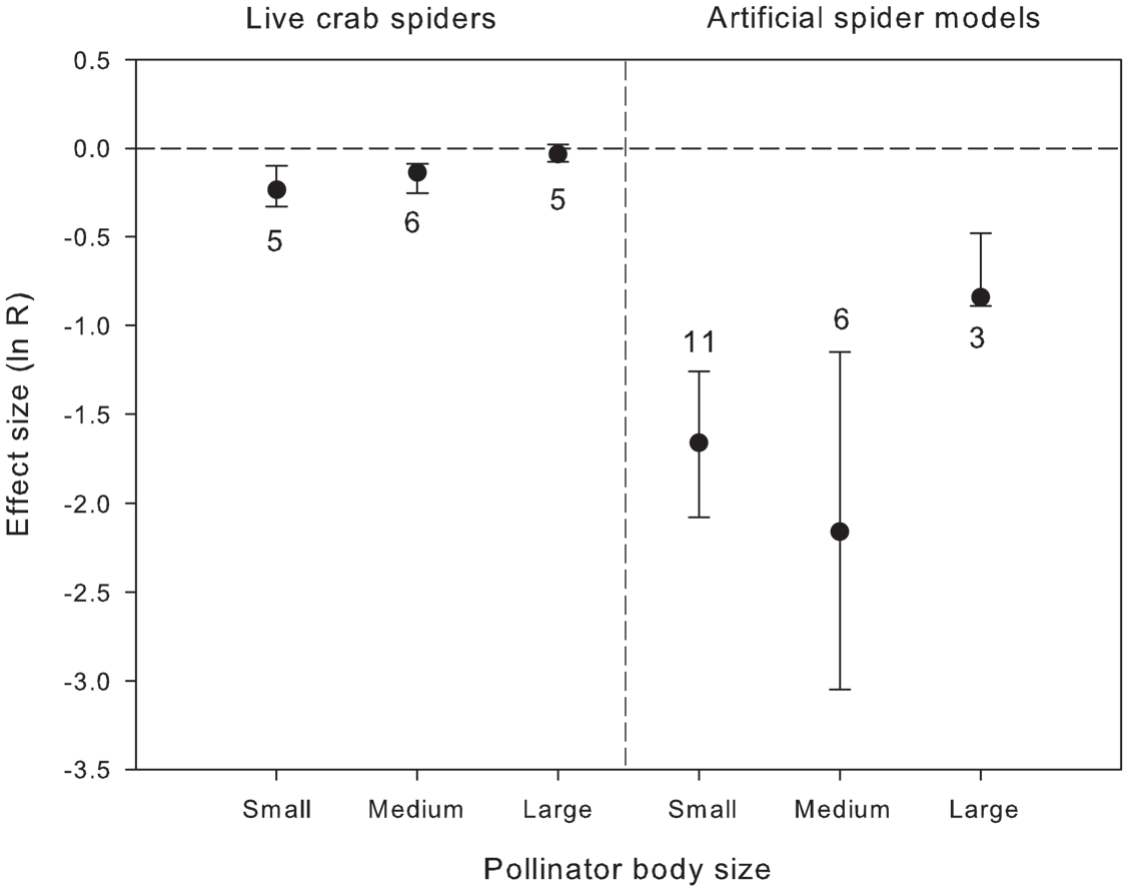
Effects (mean ln R and 95% CI) of live and artificial crab spiders on pollinators of different body size. Sample sizes are indicated next to the error bars. Negative effects indicate decrease in visitation rate on flowers with predators present; effects are considered significant if 95% CI does not include 0.

We have also examined the effect of pollinator size in studies using live and artificial crab spiders. Small and medium size arthropods decreased visitation rate by 21% and 13%, respectively, in the presence of live crab spiders, whereas large arthropods (e.g., *Bombus*, *Xylocopa*) showed no changes in flower visitation rate, i.e., confidence intervals for large pollinators do not overlap those for other prey size categories (Fig. 4). The difference among pollinator size categories was marginally significant for live predators (Qb = 0.64, P = 0.075, df = 2), but not for artificial spider models (Fig. 4, Qb = 5.24, P = 0.124, df = 2).

## Discussion

Our meta-analysis showed that predation risk has strong effects on pollinator behaviour. It significantly reduces both flower visitation rate and the time spent on flowers as well as increases flower avoidance rate. This indicates that indirect non-consumptive effects of predators on pollinators are considerable and predation risk creates the “landscape of fear” [4] for flower-visiting animals. We have also found that these anti-predatory responses were observed in most of the pollinator taxa and in response to the majority of predator taxa, suggesting that avoidance behaviour by flower-visiting animals under predation risk is widespread. With respect to sources of variation in pollinator responses to predation risk, the results of meta-analysis support three of our five original predictions. We demonstrated that live predators have weaker effects on pollinators than dead or artificial predators on flowers. We also found variation in behavioural responses among pollinator taxa, and showed that larger pollinators were less risk sensitive. Contrary to our initial expectation, predator foraging mode (sit-and-wait vs. active hunter) and pollinator lifestyle (social vs. solitary) had no significant effects on pollinator behavioural response. These results are some of the first to show that anti-predatory behaviours depend on both predator and pollinator traits. Below we discuss the likely causes of the observed differences in the predator effects as well as the implications of changes in pollinator behaviour for plant and pollinator fitness.

All predator taxa (ants, crab spiders, dragonflies, lizards, and ambush bugs) except birds had strong negative effects on visitation rate and time pollinators spent on flowers. Non-significant effect of birds could be due to small sample size (n = 3) for this taxon. The predator taxa studied here are aggressive and may feed on several pollinators per day, so they can contribute to the development of anti-predatory behaviours in flower-visiting animals. Therefore, it is expected that animals exposed to predation risk can develop mechanisms that facilitate detection and recognition of predatory traits (e.g., [14], [15], [23], [38]), even under low predation pressure [6], [7], since the costs of errors in detecting predation risk can be very high [5].

The magnitude of predator effects did not vary between predator foraging modes; we predicted that sit-and-wait predators might trigger stronger negative effects in pollinators than cues from active hunters because the former are more indicative of imminent predation risk (e.g., [26]). However, since most of the sit-and-wait predators show some degree of crypsis, allowing them to match colour backgrounds (crab spiders, ambush bugs), stronger impacts of sit-and-wait predators on pollinator behaviours may have been buffered by predator crypsis. Although recent studies have shown that pollinators avoid crab spiders on flowers independently of the colour matching on inflorescences [10], our results suggest that predator crypsis may be effective, at least to some extent. This conclusion is supported by the fact that the effects of live crab spiders on pollinators, although significant, were weaker than those of artificial crab spiders, PPE or any object on flowers. We would expect even stronger effects of live crab spiders on pollinator anti-predatory behaviours than those observed here, since experienced flower visitors can decide to leave after unsuccessful spider attacks [39] and memorise and avoid sites with higher predation risk [14], [15]. However, most of the studies on crab spiders included in the analysis (93%) were done on one species *Misumena vatia* which can change in colour (white to yellow) to match colour backgrounds of flowers on which it forages (reviewed in [28]).

Floral visitors displayed strong avoidance behaviours for objects on flowers that do not resemble predators (e.g., epoxy sphere; see Appendix S1). Similar observations have been done half a century ago by Bristowe [8], who reported that honeybees, halictid bees and syrphid flies avoided black pebbles placed on yellow dandelion flowers. These results suggest that some avoidance behaviours reported in the literature may be related to neophobia, i.e., flower visitors could avoid any structure that contrasts with flower shape or colour for reasons other than predation risk. Although considerable advances have been made towards understanding the evolution of pollinator and predator behaviours (e.g., crypsis), very few studies have attempted to understand the importance of neophobia in pollinators (e.g., [7], [9], [40]).

Pollinators belonging to the orders Squamata (geckonid lizards), Hymenoptera and Lepidoptera decreased visitation rate and time spent on flowers in the presence of predators. In contrast, predators had no effect on Diptera and Coleoptera. These results clearly indicate that pollinator behavioural responses to predation risk are taxon-specific. Even under very low probability of a gecko being preyed on by ants, they avoided flowers occupied by ants to avoid stings or to minimize resource competition. Stronger responses of Squamata support the idea that vertebrates display more aversive behaviour, since their overall visual and cognitive systems may be more complex than those of many invertebrates ([41], but see [42]), allowing a better evaluation of the foraging sites. In comparison with bees, much less is known about the sensory attributes and learning abilities that guide behaviours in insects from the orders Coleoptera, Diptera, and Lepidoptera [43]. There is evidence of high visual acuity in butterflies (reviewed by [43]), allowing them a detailed evaluation of dangerous sites on leaves [38]. Because of their thick exoskeleton, Coleoptera might be less vulnerable to predation than other orders evaluated here. As an alternative explanation, visual system in beetles seems to be less developed than in other higher order insects studied here, and they are likely to be guided primarily by olfactory cues [43]. Visual acuity in Diptera is well developed, but members of this taxon seem to depend on scent as well (review in [43]). Moreover, different groups of Diptera may vary in their ability to detect predation risk. Recent studies have shown that hoverflies display abilities to detect predation risk [10], but blowflies do not [11]. Here, Syrphidae, Sarcophagidae and Calliphoridae were not affected by predators and spent similar time on flowers (Appendix S3). Moreover, while Diptera have avoided flowers with crab spiders, they did not avoid those occupied by ants. It seems that ants may present little risk to flies; in general, ants forage on flowers to feed on nectar and pollen rather than to prey on pollinator (but see [44]). It is possible that Hymenoptera and Squamata may be avoiding flowers occupied by ants to minimize resource competition rather than avoiding predation.

Experienced workers in social hymenopterans (e.g., honeybees) can steer naïve in the colony to recruit away from dangerous flowers [9]. Thus, we expected higher avoidance of predators in social than in solitary bees, but this prediction was not supported by the results. It is possible that the ability to transmit information on predation risk to other members of the colony is restricted only to honeybees (*Apis mellifera*). On the other hand, social Hymenoptera may be under weaker selection pressure to evolve predator avoidance behaviour than solitary species because death of an individual worker in a colony has low cost for colony fitness, as compared to effects of death of a solitary bee for its individual fitness [6]. Lack of differences in predator effects on social and solitary Hymenoptera may be due to similarities in their goal-directed navigation behaviour between nest site and feeding places [45] and similar cognitive capacity and memory to evaluate foraging sites, detect predation risk and avoid being preyed on. However, even though differences between social and solitary Hymenoptera were not significant, there was a trend for solitary bees to decrease visitation rate and for social bees to decrease time spent on flowers with predators. These results might suggest some difference in foraging behaviour of social and solitary bees under predation risk that could be the topic of future research.

Pollinator size explained differences in pollinator behaviours better than pollinator lifestyle. Our analysis showed that smaller pollinators display stronger behavioural responses to predation risk than the larger species. Smaller pollinators are likely to be more vulnerable to predation because predators (e.g. crab spiders) have difficulties in capturing the larger prey [28], [39]. Thus, smaller insects might develop sensory systems to gather more precise information of the foraging sites. However, to date knowledge on variation in sensory mechanisms among smaller and larger pollinator insects is lacking and it may be a suitable theme for further studies. Previous empirical studies attempted to establish relationships between pollinator size and degree of their risk sensitivity [10], [39]. Our study is the first to present convincing results that can corroborate this assumption.

Strong effects of predation risk on pollinator behaviour detected in our meta-analysis may have important implications for both pollinator and plant fitness. Evolution of behavioural traits that confer a better ability to detect predation risk can lead to reduced probability for pollinators to be caught by predators and, ultimately, to an incremental increase in individual fitness for pollinators. Indeed, very low pollinator capture success by predators on flowers has been reported (e.g., [10], [28], [39]). On the other hand, increased avoidance and decreased visitation rate and time spent on flowers by pollinators under predation risk may decrease pollination success and, ultimately, plant fitness. A previous meta-analysis showed that when predators interfered in plant-pollinator mutualism, they decreased plant fitness by 17% [27]. Our current meta-analysis suggests that part of this effect could be due to indirect, non-consumptive interactions between pollinators and predators.

The key sources of variation associated with pollinator behaviour under predation risk were pollinator taxa and size, as well as predation risk category (live predators vs dead predators, models, objects and PPE). These results highlight the importance of simultaneously exploring the role of predator and pollinator traits in a community perspective. Although recent studies have added important knowledge on pollinator behaviour under predation risk and the role of cognitive systems, some topics still remain to be explored further. For instance, while the majority of studies have been done on Hymenoptera, behavioural responses to predation risk of other important pollinator taxa (Coleoptera, Lepidoptera, Diptera) are understudied and still poorly understood [43]. It is also largely unknown to what extent pollinator avoidance behaviour in response to predation risk is innate or acquired through learning. In addition, we found that measures of avoidance rate [23] only from unpublished data, were 4.5 times higher than measures commonly used to date (visitation rate and time spent on flowers). Avoidance behaviours seem to be a more biologically relevant measure and could be included in future studies on predator-pollinator interactions.

## Supporting Information

Appendix S1 **Material and methods for the unpublished data.**
(DOC)

Appendix S2 **List of studies used for database.**
(DOC)

Appendix S3 **Sources of variation not presented in the main text.**
(DOC)

Appendix S4 **Publication bias.**
(DOC)

Table S1 **Sources of variation and log response ratios of predator effects on visitation rate of pollinator behavior.**
(DOC)

Table S2 **Sources of variation and log response ratios of predator effects on avoidance rate of pollinator behavior.**
(DOC)

Table S3 **Sources of variation and log response ratios of predator effects on pollinator behaviour (time spent on flowers).**
(DOC)

Table S4 **Sources of variation and log response ratios of predator effects on visitation rate of pollinators of different biomass.**
(DOC)
