# Supplementary material for: A Meta-Analysis of Predation Risk Effects on Pollinator Behaviour
Source: PLoS One. 2011 Jun 13;6(6):e20689. doi: 10.1371/journal.pone.0020689 (PMC3113803; doi:10.1371/journal.pone.0020689)
Supplement: Appendix S1 — Material and methods for the unpublished data. (DOC) [file pone.0020689.s001.doc]

Appendix S1. Material and methods for the unpublished data

**Material and methods**

**a) *Study area and species***

The unpublished data on the effects of artificial predators and objects (epoxy sphere) on pollinator visitation rate were collected in four distinct geographic regions of São Paulo state, southeastern Brazil, using nine different plant species. The first area was Parque Estadual da Ilha do Cardoso (PEIC) (25°03’ S, 47°53’ W) (<http://www.cananet.com.br/peic>), an Atlantic island of 22.500 ha located in the south cost of São Paulo state, southeastern Brazil. The mean annual temperature and rainfall of the study area are 20.9 °C and 3000 mm, respectively (Barros et al. 1991). The plant species studied in this area was *Tibouchina clavata* (Melastomataceae). The second area was Reserva Biológica da Serra do Japi (23°11′S, 46°52′W; 1000 m a.s.l.) ([http://www.japi.org.br](http://www.japi.org.br/)), a semi-deciduous forest with a seasonal climate and mean monthly temperatures which vary from 13.5°C in July to 20.3°C in January (Pinto 1992). The plant species studied were *Rubus rosifolius* (Rosaceae), *Wedelia* sp.1 (Asteraceae), *Borreria verticillata* (Rubiaceae), *Manettia luteorubra* (Rubiaceae), *Tibouchina* sp. (Melastomataceae) e *Alternanthera brasiliana* (Amaranthaceae). The third study area was Núcleo Picinguaba do Parque Estadual da Serra do Mar (23º40’S, 44º45’W) (<http://www.iflorestsp.br/picinguaba.htm>), Ubatuba city, a reserve of *ca*. 47.500 ha of Atlantic Rainforest, without dry season, and with mean annual temperature of 22.6ºC (CEPAGRI). The species studied was *Wedelia paludosa* (Asteraceae). The fourth study area was the campus of the State University of São Paulo (UNESP), São José do Rio Preto city ([http://www.ibilce.unesp.br](http://www.ibilce.unesp.br/)). The plant species studied was *Wedelia* sp. 2 (Asteraceae).

**b) *Experimental design***

Detailed experimental design used to test the effects of artificial predators and any object on flowers on the visitation and avoidance rate of floral visitors in the nine plant species studied here is previously described in Gonçalves-Souza et al. (2008) for *Rubus rosifolius*; here we present only general methods and procedures that differed from those described earlier. We conducted a randomized-block experiment, each block consisting of three flowers, each one in different individual plant, which were randomly designated to receive one of the following treatments: (i) an artificial crab spider (models), (ii) a sphere, and (iii) control (an empty flower). Figure 1 below illustrates the treatments. Sample sizes for each plant species are presented in the Appendix C. The artificial spiders were made using epoxy resin (prosoma and opistosoma), with two open metal staples fixed at the base of prosoma to imitate the two forelimbs (see Fig. 1). An acrylic dye (straw color) was used to paint the spiders, thus making them similar in color to common crab spider of the genus *Misumenops* (Thomisidae). The models were constructed and painted 10 years before the experiments, so any colour scent was eliminated. The sphere (from the treatment ii) was the same as opistosoma from the spider model painted as described above. The artificial spiders and sphere were placed on the petals of the flower (as in Suttle 2003, fig. 1).

The flowers of each experimental block were at least 30 cm apart; they were observed simultaneously during four periods of 40 min each on the same day, with intervals of 10 min between sections. The number of visits by pollinators and avoidance rate were recorded for each section, but grouped together for the analyses (replicate: total/flower = 160 min). An exception to the general procedure described above was *Wedelia paludosa* (Picinguaba), in which we did observations in intervals of one hour. The period of observations was typically from 8:00 am to 6:00 pm. Pollinators were considered visiting the flowers if they landed on the flower and stayed there for at least 3 s and avoiding the flowers when they approach the flower but instead of landing switch to another flower or leave the area (Gonçalves-Souza et al. 2008).

**REFERENCES**

Barros, F., Melo, M.M.R.F., Chiea, S.A.C., Kirizawa, M., Wanderley, M.G.L. & Jung-Mendaçolli, S.L. 1991. Flora fanerogâmica da Ilha do Cardoso, I. Instituto de Botânica, São Paulo.

Gonçalves-Souza, T., Omena, P.M., Souza, J.C. & Romero, G.Q. 2008. Trait-mediated effects on flowers: artificial spiders deceive pollinators and decrease plant fitness. Ecology 89: 2407-2413.

Pinto HS (1992) Clima na Serra do Japi. In: Morellato LPC (org) História natural da Serra do Japi: ecologia e preservação de uma área florestal no Sudeste do Brasil. Editora da Unicamp, Campinas, pp 30–39

Suttle, K. B. 2003. Pollinators as mediators of top-down effects on plants. Ecology Letters 6:688–694.

Fig. 1. (a) Artificial spider, (b) sphere (spider abdomen) and a *Pseudoscada erruca* butterfly (Ithomiinae, Nymphalidae), and (c) control and *Trigona* sp. (Apidae) on flowers of *Rubus rosifolius* (Rosaceae) at Serra do Japi, southeastern Brazil.
