# Supplementary material for: A Meta-Analysis of Predation Risk Effects on Pollinator Behaviour
Source: PLoS One. 2011 Jun 13;6(6):e20689. doi: 10.1371/journal.pone.0020689 (PMC3113803; doi:10.1371/journal.pone.0020689)
Supplement: Appendix S2 — List of studies used for database. (DOC) [file pone.0020689.s002.doc]

Appendix S2. List of studies used for database

Visitation rate

Abbott, K. R. 2006. Bumblebees avoid flowers containing evidence of past predation event. Canadian Journal of Zoology 84: 1240-1247.

Abbott, K. R. & Dukas, R. 2009. Honeybees consider flower danger in their waggle dance. Animal Behaviour 78: 633–635.

Agarwal, V.M. & Rastogi, N. 2008. Role of floral repellents in the regulation of flower visits of extrafloral nectary-visiting ants in an Indian crop plant. Ecological Entomology 33: 59–65.

Altshuler, D.L. 1999. Novel interactions of non-pollinating ants with pollinators and fruit consumers in a tropical forest. Oecologia 119: 600-606.

Ashman, T-L & king, E.A. 2005. Are flower-visiting ants mutualists or antagonists? A study in a gynodioecious wild strawberry. American Journal of Botany 92: 891–895.

Blancafort, X. & Gómez, C. 2005. Consequences of the Argentine ant, *Linepithema humile* (Mayr), invasion on pollination of *Euphorbia characias* (L.) (Euphorbiaceae). Acta Oecologica 28: 49–55.

Brechbühl, R., Casas, J. & Bacher, S. 2010a. Ineffective crypsis in a crab spider: a prey community perspective. Proceedings of the Royal Society of London B 277: 739-746.

Brechbühl, R., Kropf, C. & Bacher, S. 2010b. Impact of flower-dwelling crab spiders on plant-pollinator mutualisms. Basic and Applied Ecology 11: 76-82.

Dukas, R. 2005. Bumble bee predators reduce pollinator density and plant fitness. Ecology 86: 1401-1406.

Dukas, R. & Morse, D.H. 2003. Crab spiders affect flower visitation by bees. Oikos 101: 157-163.

Dukas, R. & Morse, D.H. 2005. Crab spiders show mixed effects on flower-visiting bees and no effect on plant fitness components. Ecoscience 12: 244–247.

Elliott, N.B. & Elliott, W.M. 1991. Effect of an ambush predator, *Phymata americana* Melin, on behavior of insects visiting *Daucus carota*. American Midland Naturalist 126: 198-202.

Elliott, N.B. & Elliott, W.M. 1994. Recognition and avoidance of the predator *Phymata americana* Melin on *Solidago odora* Ait. by late season floral visitors. American Midland Naturalist 131: 378-380.

Gonçalves-Souza, T., Omena, P.M., Souza, J.C. & Romero, G.Q. 2008. Trait-mediated effects on flowers: artificial spiders deceive pollinators and decrease plant fitness. Ecology 89: 2407-2413.

Hansen D.M. & Müller, C.B. 2009. Invasive ants disrupt Gecko pollination and seed dispersal of the endangered plant *Roussea simplex* in Mauritius. Biotropica 41: 202–208.

Junker, R., Chung, A.Y.C. & Blüthgen, N. 2007. Interaction between flowers, ants and pollinators: additional evidence for floral repellence against ants. Ecological Research 22: 665–670.

Knight, T.M., McCoy, M.W., Chase, J.M., McCoy, K.A. & Holt, R.D. 2005. Trophic cascades across ecosystems. Nature 437:880-883.

Lima, S.L. 1991. Energy, predators and the behaviour of feeding hummingbirds. Evolutionary Ecology 5: 220–230.

Muñoz, A.A. & Arroyo, M.T.K. 2004. Negative impacts of a vertebrate predator on insect pollinator visitation and seed output in *Chuquiraga oppositifolia*, a high Andean shrub. Oecologia 138: 66–73.

Norment, C.J. 1988. The effect of nectar-thieving ants on the reproductive success of *Frasera speciosa* (Gentianaceae). American Midland Naturalist 120: 331-336.

Robertson, I.C. & Maguire, D.K. 2005. Crab spiders deter insect visitations to slickspot peppergrass flowers. Oikos 109: 577-582.

Suttle, K. B. 2003. Pollinators as mediators of top-down effects on plants. Ecology Letters 6:688–694.

Tsuji, K, Hasyim, A., Harlion & Nakamura, K. 2004. Asian weaver ants, *Oecophylla smaragdina*, and their repelling of pollinators. Ecological Research 19: 669–673.

Foraging time

Abbott, K. R. 2006. Bumblebees avoid flowers containing evidence of past predation event. Canadian Journal of Zoology 84: 1240-1247.

Blancafort, X. & Gómez, C. 2005. Consequences of the Argentine ant, *Linepithema humile* (Mayr), invasion on pollination of *Euphorbia characias* (L.) (Euphorbiaceae). Acta Oecologica 28: 49–55.

Elliott, N.B. & Elliott, W.M. 1991. Effect of an ambush predator, *Phymata americana* Melin, on behavior of insects visiting *Daucus carota*. American Midland Naturalist 126: 198-202.

Elliott, N.B. & Elliott, W.M. 1994. Recognition and avoidance of the predator *Phymata americana* Melin on *Solidago odora* Ait. by late season floral visitors. American Midland Naturalist 131: 378-380.

Hansen D.M. & Müller, C.B. 2009. Invasive ants disrupt Gecko pollination and seed dispersal of the endangered plant *Roussea simplex* in Mauritius. Biotropica 41: 202–208.

Junker, R., Chung, A.Y.C. & Blüthgen, N. 2007. Interaction between flowers, ants and pollinators: additional evidence for floral repellence against ants. Ecological Research 22: 665–670.

Lach, L. 2008a. Argentine ants displace floral arthropods in a biodiversity hotspot. Diversity and Distributions 14: 281–290.

Lach, L. 2008b. Floral visitation patterns of two invasive ant species and their effects on other hymenopteran visitors. Ecological Entomology 33: 155–160.

Lima, S.L. 1991. Energy, predators and the behaviour of feeding hummingbirds. Evolutionary Ecology 5: 220–230.

Muñoz, A.A. & Arroyo, M.T.K. 2004. Negative impacts of a vertebrate predator on insect pollinator visitation and seed output in *Chuquiraga oppositifolia*, a high Andean shrub. Oecologia 138: 66–73.

Ness, J.H. 2006. A mutualism’s indirect costs: the most aggressive plant bodyguards also deter pollinators. Oikos 113: 506-514.

Suttle, K. B. 2003. Pollinators as mediators of top-down effects on plants. Ecology Letters 6:688–694.

Floral visitor size

Brechbühl, R., Casas, J. & Bacher, S. 2010a. Ineffective crypsis in a crab spider: a prey community perspective. Proceedings of the Royal Society of London B 277: 739-746.

Dukas, R. & Morse, D.H. 2003. Crab spiders affect flower visitation by bees. Oikos 101: 157-163.

Dukas, R. & Morse, D.H. 2005. Crab spiders show mixed effects on flower-visiting bees and no effect on plant fitness components. Ecoscience 12: 244–247.
