# Supplementary material for: A Meta-Analysis of Predation Risk Effects on Pollinator Behaviour
Source: PLoS One. 2011 Jun 13;6(6):e20689. doi: 10.1371/journal.pone.0020689 (PMC3113803; doi:10.1371/journal.pone.0020689)
Supplement: Appendix S3 — Sources of variation not presented in the main text. (DOC) [file pone.0020689.s003.doc]

Appendix S3. Sources of variation not presented in the main text

There was no significant difference between responses of pollinator families and subfamilies to live predators (visitation rate: Qb = 11.2, P = 0.167; time spent on flowers: Qb = 24.2, P = 0.105). Presence of predators significantly decreased visitation rates by all pollinator taxa except Syrphidae and Satirinae (Lepidoptera) (Fig. 1a) and reduced time spent on flowers by all pollinator families except Syrphidae, Calliphoridae and Sarcophagidae (Diptera) (Fig. 1b).

There was no significant difference between effects of native and invasive ants on pollinator visitation rate (native: ln R = -0.59, 95% CI = -1.01 to -0.29, n = 10; invasive: ln R = -0.40, 95% CI = -1.26 to 0.28, n = 7; Qb =0.26, P = 0.66) and time spent on flowers (native: ln R = -0.68, 95% CI = -1.37 to 0.16, n = 4; invasive: ln R = -0.67, 95% CI = -1.41 to -0.08, n = 10; Qb = 0.0005, P = 0.99).

The overall effects of presence of live predators on pollinator visitation rates did not differ between experimental and observational studies (experimental: lnR = -0.46, 95% CI = -0.72 to -0.27, n = 21; observational: lnR = -0.41, 95% CI = -0.73 to -0.15, n = 26, Qb = 0.07, P = 0.80, df = 1). The same was true for effects on time spent on flowers (experimental: ln R = -0.84, 95% CI =-1.36 to -0.40, n = 14; observational: ln R = -0.62, 95% CI = -1.10 to -0.27, n = 20; Qb = 0.36, P = 0.54, df = 1).

Fig. 1. Effects (mean ln R and 95% CI) of live predators on visitation rate (a) and time spent (b) by flower-visiting animals of diverse taxa (family and subfamily) on flowers. Sample sizes are indicated next to the error bars. API = Apidae, SYR = Syrphidae, HAL = Halictidae, VES = Vespidae, GEK = Gekkonidae, SAT = Satirinae, AND = Andrenidae, CAL = Calliphoridae, SAR = Sarcophagidae. N/A = data not available.
