# Supplementary material for: A Meta-Analysis of Predation Risk Effects on Pollinator Behaviour
Source: PLoS One. 2011 Jun 13;6(6):e20689. doi: 10.1371/journal.pone.0020689 (PMC3113803; doi:10.1371/journal.pone.0020689)
Supplement: Appendix S4 — Publication bias. (DOC) [file pone.0020689.s004.doc]

Appendix S4. Publication bias

**Material and methods**

We tested for publication bias in our dataset by using the funnel plot technique, in which effect sizes are plotted against their sample sizes. Bias in our data set would be detected if the funnel plot is asymmetrical or presents gaps near the null effects (Rosenberg et al. 2000). We have also calculated rank correlations between effect size and sample size to test for publication bias on visitation rate and time spent variables. We have also tested for robustness of our results against potential publication bias by calculating weighted fail-safe numbers (Rosenberg 2005) using the Fail-Safe Number Calculator ([www.rosenberglab.net/software.php#failsafe](http://www.rosenberglab.net/software.php#failsafe)). This estimates the number of unpublished studies weith non-significant results that would have to be included in the meta-analysis to change the results from significant to non-significant. We used fixed effect models since for some comparisons the model for random effects collapsed to fixed effect. A t-test based on normal distribution was used to test if the mean effect size differs from the null model (Rosenberg 2005).

**Results and Discussion**

Funnel plots and rank correlations indicated that published studies with smaller sample sizes tended to report stronger negative effects of predators on visitation rate (Figure 1). This is unlikely to be the result of publication bias against small and nonsignificant effects because similar pattern occurred in unpublished data (Figure 2). Instead, it probably indicates that studies with small sample sizes are just more likely to produce extreme results than studies with larger sample sizes. The majority of studies had sample sizes <30. When we removed from the analyses few studies with sample size >30, rank correlations between sample size and effect size became not significant (Figure 3). There was no correlation between sample size and effect size for time spent (Fig. 4). The weighted fail-safe numbers (t-N+) for the effects of predators on visitation rate without unpublished data, on visitation rate including unpublished data, and time spent on flowers showed, respectively, that 1297, 4693 and 4164 non-significant studies would have to be added to the analysis to change the statistical significance of the observed effects; the mean effect sizes for the estimates above differed from the null model (t-test; P<0.0001). This indicates that even if the publication bias against small and nonsignificant effects occurs, the results of our meta-analysis are robust and unlikely to be influenced by publication bias.

**Literature cited**

Rosenberg, M. S. 2005. The file-drawer problem revisited: A general weighted method for calculating fail-safe numbers in meta-analysis. [Evolution](http://lifesciences.asu.edu/evolution/) 59:464-468.

Rosenberg, M. S., Adams, D. C. & Gurevitch, J. 2000. MetaWin: statistical software for meta-analysis. Version 2.0. Sinauer Associates, Inc., Sunderland, Massachusetts.


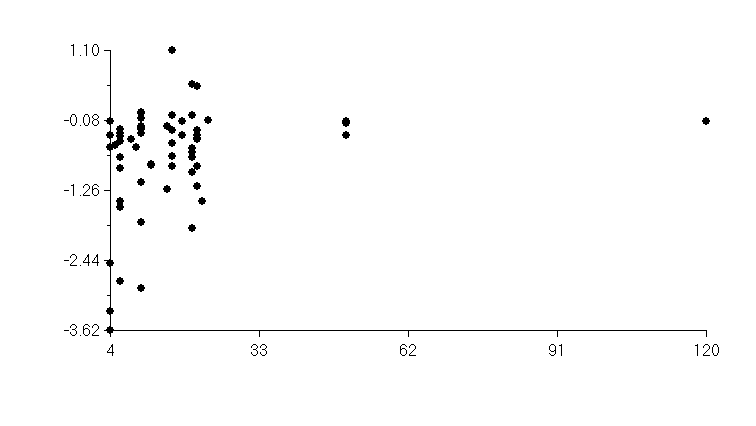


Effect size (ln R)

Sample size (n)

Figure 1. Funnel plot of effect size (ln R) for pollinator visitation rate (only published data) against sample size (Kendall’s Tau: Tau = 0.228, Z = 2.61, P = 0.009; Spearman rank: r^2^ = 0.29, P = 0.020).


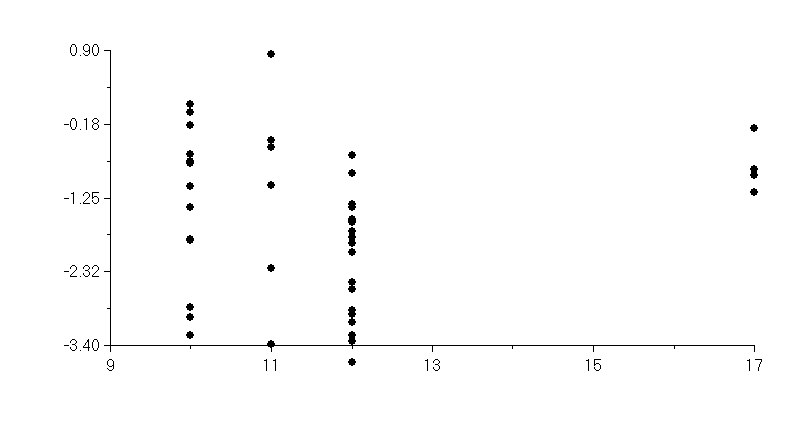


Effect size (ln R)

Sample size (n)

Figure 2. Funnel plot of effect size (ln R) for pollinator visitation rate (only unpublished data) against sample size. (Kendall’s Tau: Tau = -0.123, Z = -1.18, P = 0.24; Spearman rank: r^2^ = -0.17, P = 0.27)


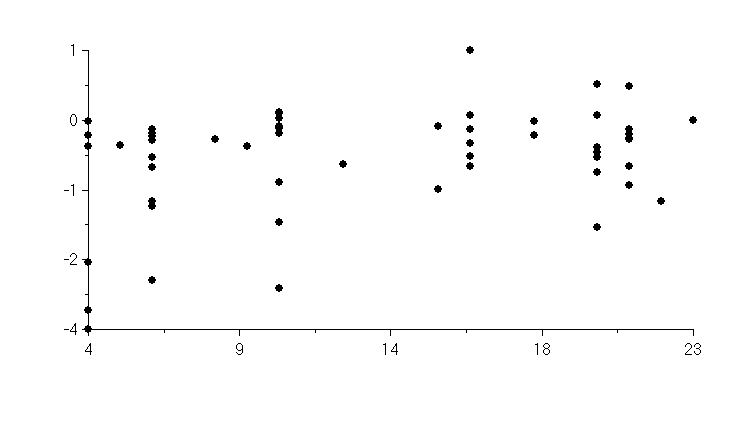


Effect size (ln R)

Sample size (n)

Figure 3. Funnel plot of effect size (ln R) for pollinator visitation rate (only published data) against sample size, with outliers (i.e., sample size > 30) removed (Kendall’s Tau: Tau = 0.153, Z = 1.69, P = 0.09; Spearman rank: r^2^ = 0.19, P = 0.145).


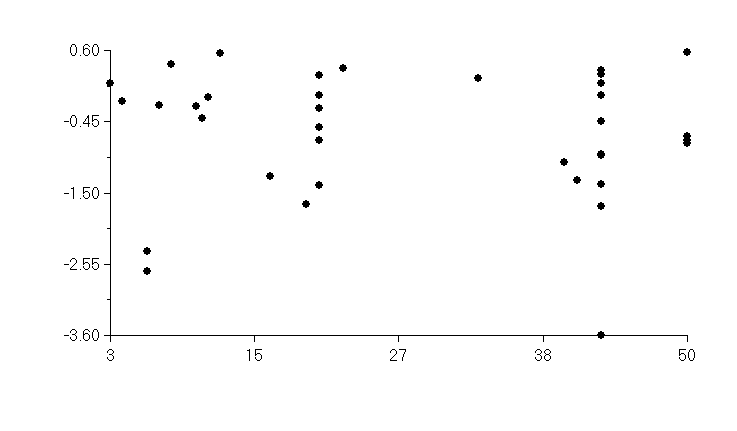


Effect size (ln R)

Sample size (n)

Figure 4. Funnel plot of effect size (ln R) for time spent on flowers (only published data) against sample size (Kendall’s Tau: Tau = -0.043, Z = -0.37, P = 0.71; Spearman rank: r^2^ = -0.07, P = 0.67).
