# Supplementary material for: A Meta-Analysis of Predation Risk Effects on Pollinator Behaviour
Source: PLoS One. 2011 Jun 13;6(6):e20689. doi: 10.1371/journal.pone.0020689 (PMC3113803; doi:10.1371/journal.pone.0020689)
Supplement: Table S1 — Sources of variation and log response ratios of predator effects on visitation rate of pollinator behavior. (DOC) [file pone.0020689.s005.doc]

Table S1. Sources of variation and log response ratios of predator effects on visitation rate of pollinator behaviour

|  |  |  | Predators | |  |  |  |  | Floral visitors | | |  | Log response ratio | |  |
| --- | --- | --- | --- | --- | --- | --- | --- | --- | --- | --- | --- | --- | --- | --- | --- |
| References and data source | Taxa | Category ^a^ | | Hunting Mode | Native/Invas ^b^ |  | Family | | | Order | Solit/Social |  | Effect size | Variance | |
| Abbott 2006; fig.1 | ... | PPEv | | ... | ... |  | Apidae | | | Hym | Social |  | -0.8272 | 0.1452 | |
| Abbott 2006; fig.1 | ... | PPEv | | ... | ... |  | Apidae | | | Hym | Social |  | -1.4503 | 0.1479 | |
| Abbott 2006; fig.1 | ... | PPEs | | ... | ... |  | Apidae | | | Hym | Social |  | -0.3337 | 0.0558 | |
| Abbott & Dukas 2009; text | ... | PPEv | | ... | ... |  | Apidae | | | Hym | Social |  | -0.5059 | 0.0575 | |
| Agarwal & Rastogi 2008; text | Ant | Live | | Hunter | Nat |  | Several | | | Several | ... |  | -0.1029 | 0.0212 | |
| Altshuler 1999; table 3 | Ant | Live | | Hunter | Nat |  | Several | | | Several | ... |  | -1.2479 | 0.3941 | |
| Ashman & King 2005; fig. 1B | Ant | Live | | Hunter | Nat |  | Several | | | Several | ... |  | 0.0308 | 0.3185 | |
| Ashman & King 2005; fig. 1B | Ant | Live | | Hunter | Nat |  | Several | | | Several | ... |  | -0.2063 | 0.026 | |
| Blancafort & Gómez 2005; table 2 | Ant | Live | | Hunter | Inv |  | Calliphoridae | | | Dip | ... |  | -0.2412 | 0.0511 | |
| Blancafort & Gómez 2005; table 2 | Ant | Live | | Hunter | Inv |  | Syrphidae | | | Dip | ... |  | 0 | 0.2125 | |
| Blancafort & Gómez 2005; table 2 | Ant | Live | | Hunter | Inv |  | Tachinidae | | | Dip | ... |  | 0 | 0.125 | |
| Blancafort & Gómez 2005; table 2 | Ant | Live | | Hunter | Inv |  | Sarcophagidae | | | Dip | ... |  | 1.0986 | 0.0903 | |
| Blancafort & Gómez 2005; table 2 | Ant | Live | | Hunter | Inv |  | Muscidae | | | Dip | ... |  | -0.6931 | 0.6406 | |
| Brechbühl et al. 2010a, fig. 1 | Crab spider | Live | | Sit-and-wait | Nat |  | Colletidae | | | Hym | Solitary |  | -0.2245 | 0.1124 | |
| Brechbühl et al. 2010a, fig. 1 | Crab spider | Live | | Sit-and-wait | Nat |  | Halictidae | | | Hym | Solitary |  | -0.1835 | 0.1284 | |
| Brechbühl et al. 2010a, fig. 1 | Crab spider | Live | | Sit-and-wait | Nat |  | Apidae | | | Hym | Social |  | -0.0445 | 0.1958 | |
| Brechbühl et al. 2010a, fig. 1 | Crab spider | Live | | Sit-and-wait | Nat |  | Syrphidae | | | Dip | ... |  | -0.1922 | 0.2416 | |
| Brechbühl et al. 2010b; fig. 1a-b | Crab spider | Model | | Sit-and-wait | ... |  | Apidae | | | Hym | Social |  | -0.2995 | 0.1896 | |
| Brechbühl et al. 2010b; fig. 1a-b | Crab spider | Dead | | Sit-and-wait | Nat |  | Apidae | | | Hym | Social |  | -0.237 | 0.1806 | |
| Brechbühl et al. 2010b; fig. 1c | Crab spider | Model | | Sit-and-wait | ... |  | ? | | | Hym | Solitary |  | -0.3472 | 0.0666 | |
| Brechbühl et al. 2010b; fig. 1c | Crab spider | Dead | | Sit-and-wait | Nat |  | ? | | | Hym | Solitary |  | -0.7098 | 0.1212 | |
| Brechbühl et al. 2010b; fig. 1d | Crab spider | Model | | Sit-and-wait | ... |  | Syrphidae | | | Dip | ... |  | -0.4281 | 0.096 | |
| Brechbühl et al. 2010b; fig. 1d | Crab spider | Dead | | Sit-and-wait | Nat |  | Syrphidae | | | Dip | ... |  | -1.4489 | 0.2454 | |
| Dukas 2005; fig. 3a | Wasp | Live | | Hunter | Nat |  | Apidae | | | Hym | Social |  | -0.8857 | 0.2043 | |
| Dukas & Morse 2003; fig. 2 | Crab spider | Live | | Sit-and-wait | Nat |  | Apidae | | | Hym | Social |  | -0.0857 | 0.1243 | |
| Dukas & Morse 2005; fig. 1 | Crab spider | Live | | Sit-and-wait | Nat |  | Apidae | | | Hym | Social |  | -0.3081 | 0.0516 | |
| Elliott & Elliott 1991; table 1 | Phymatid | Live | | Sit-and-wait | Nat |  | Several | | | Several | ... |  | -0.1049 | 0.0644 | |
| Elliott & Elliott 1994; table 1 | Phymatid | Live | | Sit-and-wait | Nat |  | Several | | | Several | ... |  | -0.3253 | 0.0338 | |
| Elliott & Elliott 1994; table 2 | Phymatid | Live | | Sit-and-wait | Nat |  | Apidae | | | Hym | Social |  | -0.1275 | 0.0386 | |
| Elliott & Elliott 1994; table 3 | Phymatid | Live | | Sit-and-wait | Nat |  | Vespidae | | | Hym | Social |  | -0.0928 | 0.0742 | |
| Gonçalves-Souza et al. 2008; fig. 3a | Crab spider | Model | | Sit-and-wait | ... |  | Several | | | Hym | Social |  | -1.8021 | 0.1415 | |
| Gonçalves-Souza et al. 2008; fig. 3a | ... | Object | | ... | ... |  | Several | | | Hym | Social |  | 0.0503 | 0.1148 | |
| Gonçalves-Souza et al. 2008; fig. 3a | Crab spider | Model | | Sit-and-wait | ... |  | Several | | | Lep | ... |  | -2.9178 | 0.689 | |
| Gonçalves-Souza et al. 2008; fig. 3a | ... | Object | | ... | ... |  | Several | | | Lep | ... |  | -1.126 | 0.6068 | |
| Hansen & Müller 2009; fig. 2a | Ant | Live | | Hunter | Inv |  | Gekkonidae | | | Squamata | ... |  | -2.7872 | 0.6311 | |
| Hansen & Müller 2009; fig. 2a | Ant | Live | | Hunter | Inv |  | Gekkonidae | | | Squamata | ... |  | -1.5471 | 0.1075 | |
| Junker et al. 2007; text | Ant | Live | | Hunter | Nat |  | Apidae | | | Hym | Social |  | -0.4055 | 0.2118 | |
| Junker et al. 2007; text | Ant | Live | | Hunter | Nat |  | Apidae | | | Hym | Social |  | -0.1823 | 0.0536 | |
| Junker et al. 2007; text | Ant | Live | | Hunter | Nat |  | Apidae | | | Hym | Social |  | -0.5293 | 0.1299 | |
| Knight et al. 2005; fig. S1 | Dragonfly | Live | | Hunter | Nat |  | Arctiidae | | | Lep | ... |  | -0.3352 | 0.2899 | |
| Knight et al. 2005; fig. S1 | Dragonfly | Live | | Hunter | Nat |  | Halictidae | | | Hym | Solitary |  | -3.2958 | 4.4444 | |
| Knight et al. 2005; fig. S3 | Dragonfly | Live | | Hunter | Nat |  | Several | | | Dip | ... |  | -0.5274 | 0.1542 | |
| Knight et al. 2005; fig. S3 | Dragonfly | Live | | Hunter | Nat |  | Noctuidae | | | Lep | ... |  | -2.4819 | 0.7674 | |
| Knight et al. 2005; fig. S3 | Dragonfly | Live | | Hunter | Nat |  | Halictidae | | | Hym | Solitary |  | -3.6185 | 0.5568 | |
| Lima 1991; fig. 2b | ... | PR | | ... | ... |  | Trochilidae | | | Trochiliformes | ... |  | -0.0999 | 0.0215 | |
| Munõz & Arroyo 2004; fig. 2 | Bird | Live | | Hunter | Nat |  | Satiridae | | | Lep | ... |  | 0.0068 | 0.223 | |
| Munõz & Arroyo 2004; fig. 2 | Lizard | Live | | Sit-and-wait | Nat |  | Satiridae | | | Lep | ... |  | -0.5488 | 0.2656 | |
| Munõz & Arroyo 2004; fig. 2 | Bird | Live | | Hunter | Nat |  | Syrphidae | | | Dip | ... |  | 0.5198 | 0.4072 | |
| Munõz & Arroyo 2004; fig. 2 | Lizard | Live | | Sit-and-wait | Nat |  | Syrphidae | | | Dip | ... |  | -0.6259 | 0.8265 | |
| Munõz & Arroyo 2004; fig. 2 | Bird | Live | | Hunter | Nat |  | Andrenidae | | | Hym | Solitary |  | -0.7072 | 0.2962 | |
| Munõz & Arroyo 2004; fig. 2 | Lizard | Live | | Sit-and-wait | Nat |  | Andrenidae | | | Hym | Solitary |  | -0.9583 | 0.2909 | |
| Norment 1988; table 2 | Ant | Live | | Hunter | Nat |  | Several | | | Several | ? |  | -0.8357 | 0.0454 | |
| Robertson & Maguire 2005; table 2b | Crab spider | Live | | Sit-and-wait | Nat |  | Halictidae | | | Hym | Solitary |  | -0.2412 | 0.1203 | |
| Robertson & Maguire 2005; table 2b | Crab spider | Live | | Sit-and-wait | Nat |  | Sphecidae | | | Hym | Solitary |  | -1.1872 | 0.4884 | |
| Robertson & Maguire 2005; table 2b | Crab spider | Live | | Sit-and-wait | Nat |  | Vespidae | | | Hym | Social |  | -0.3254 | 0.7892 | |
| Robertson & Maguire 2005; table 2b | Crab spider | Live | | Sit-and-wait | Nat |  | Gelechiidae | | | Lep | ... |  | -0.8575 | 0.8775 | |
| Robertson & Maguire 2005; table 2b | Crab spider | Live | | Sit-and-wait | Nat |  | Merylidae | | | Col | ... |  | -0.3836 | 0.2124 | |
| Robertson & Maguire 2005; table 2b | Crab spider | Live | | Sit-and-wait | Nat |  | Bombyliidae | | | Dip | ... |  | -0.4055 | 0.5775 | |
| Robertson & Maguire 2005; table 2b | Crab spider | Live | | Sit-and-wait | Nat |  | Several | | | Several | ? |  | 0.494 | 0.5254 | |
| Suttle 2003; fig 2, text | Crab spider | Live | | Sit-and-wait | Nat |  | Several | | | Several | ? |  | -0.4726 | 0.0417 | |
| Tsuji et al 2004; fig. 2 | Ant | Live | | Hunter | Nat |  | Apidae | | | Hym | Social |  | -1.8971 | 0.1175 | |
| Tsuji et al 2004; fig. 2 | Ant | Live | | Hunter | Nat |  | Several | | | Several | ? |  | -0.8622 | 0.5092 | |
| Unpubl. data (*Rubus rosifolius*) | Crab spider | Model | | Sit-and-wait | ... |  | Halictidae | | | Hym | Solitary |  | -1.8718 | 1.5228 | |
| Unpubl. data (*Rubus rosifolius*) | Crab spider | Object | | Sit-and-wait | ... |  | Halictidae | | | Hym | Solitary |  | -0.619 | 1.5228 | |
| Unpubl. data (*Rubus rosifolius*) | Crab spider | Model | | Sit-and-wait | ... |  | Apidae | | | Hym | Social |  | -0.7419 | 0.2103 | |
| Unpubl. data (*Rubus rosifolius*) | Crab spider | Object | | Sit-and-wait | ... |  | Apidae | | | Hym | Social |  | 0.1174 | 0.1636 | |
| Unpubl. data (*Rubus rosifolius*) | Crab spider | Model | | Sit-and-wait | ... |  | ? | | | Hym | ? |  | -1.3863 | 0.8289 | |
| Unpubl. data (*Rubus rosifolius*) | Crab spider | Object | | Sit-and-wait | ... |  | ? | | | Hym | ? |  | 0 | 1.172 | |
| Unpubl. data (*Rubus rosifolius*) | Crab spider | Model | | Sit-and-wait | ... |  | Nymphalidae | | | Lep | ... |  | -2.9957 | 1.416 | |
| Unpubl. data (*Rubus rosifolius*) | Crab spider | Object | | Sit-and-wait | ... |  | Nymphalidae | | | Lep | ... |  | -0.1924 | 1.0658 | |
| Unpubl. data (*Wedelia* sp. 1) | Crab spider | Model | | Sit-and-wait | ... |  | Apidae | | | Hym | Social |  | -1.7399 | 0.5686 | |
| Unpubl. data (*Wedelia* sp. 1) | Crab spider | Object | | Sit-and-wait | ... |  | Apidae | | | Hym | Social |  | -1.5989 | 0.7618 | |
| Unpubl. data (*Wedelia* sp. 1) | Crab spider | Model | | Sit-and-wait | ... |  | Vespidae | | | Hym | Social |  | -2.5829 | 0.63 | |
| Unpubl. data (*Wedelia* sp. 1) | Crab spider | Object | | Sit-and-wait | ... |  | Vespidae | | | Hym | Social |  | -1.9196 | 0.7975 | |
| Unpubl. data (*Wedelia* sp. 1) | Crab spider | Model | | Sit-and-wait | ... |  | Megachilidae | | | Hym | Solitary |  | -0.8883 | 0.4055 | |
| Unpubl. data (*Wedelia* sp. 1) | Crab spider | Object | | Sit-and-wait | ... |  | Megachilidae | | | Hym | Solitary |  | -0.6313 | 0.4327 | |
| Unpubl. data (*Wedelia* sp. 1) | Crab spider | Model | | Sit-and-wait | ... |  | Nymphalidae | | | Lep | ... |  | -1.5999 | 0.7124 | |
| Unpubl. data (*Wedelia* sp. 1) | Crab spider | Object | | Sit-and-wait | ... |  | Nymphalidae | | | Lep | ... |  | -1.3486 | 0.6794 | |
| Unpubl. data (*Wedelia* sp. 1) | Crab spider | Model | | Sit-and-wait | ... |  | Syrphidae | | | Dip | ... |  | -2.0408 | 0.4008 | |
| Unpubl. data (*Wedelia* sp. 1) | Crab spider | Object | | Sit-and-wait | ... |  | Syrphidae | | | Dip | ... |  | -1.8238 | 0.3099 | |
| Unpubl. data (*Wedelia* sp. 2) | Crab spider | Model | | Sit-and-wait | ... |  | Apidae | | | Hym | Social |  | -2.4889 | 1.1237 | |
| Unpubl. data (*Wedelia* sp. 2) | Crab spider | Object | | Sit-and-wait | ... |  | Apidae | | | Hym | Social |  | -1.3863 | 0.6189 | |
| Unpubl. data (*Wedelia* sp. 2) | Crab spider | Model | | Sit-and-wait | ... |  | Halictidae | | | Hym | Solitary |  | -2.9463 | 1.0622 | |
| Unpubl. data (*Wedelia* sp. 2) | Crab spider | Object | | Sit-and-wait | ... |  | Halictidae | | | Hym | Solitary |  | -1.5661 | 0.3682 | |
| Unpubl. data (*Borreria verticillata*) | Crab spider | Model | | Sit-and-wait | ... |  | Apidae | | | Hym | Social |  | -3.2581 | 0.3432 | |
| Unpubl. data (*Borreria verticillata*) | Crab spider | Object | | Sit-and-wait | ... |  | Apidae | | | Hym | Social |  | -3.6438 | 1.0432 | |
| Unpubl. data (*Borreria verticillata*) | Crab spider | Model | | Sit-and-wait | ... |  | Vespidae | | | Hym | Social |  | -2.8944 | 1.1599 | |
| Unpubl. data (*Borreria verticillata*) | Crab spider | Object | | Sit-and-wait | ... |  | Vespidae | | | Hym | Social |  | -2.8944 | 1.1941 | |
| Unpubl. data (*Borreria verticillata*) | Crab spider | Model | | Sit-and-wait | ... |  | Syrphidae | | | Dip | ... |  | -3.3476 | 0.7737 | |
| Unpubl. data (*Borreria verticillata*) | Crab spider | Object | | Sit-and-wait | ... |  | Syrphidae | | | Dip | ... |  | -3.0659 | 0.5993 | |
| Unpubl. data (*Tibouchina clavata*) | Crab spider | Model | | Sit-and-wait | ... |  | Halictidae | | | Hym | Solitary |  | -0.2412 | 0.6979 | |
| Unpubl. data (*Tibouchina clavata*) | Crab spider | Object | | Sit-and-wait | ... |  | Halictidae | | | Hym | Solitary |  | -1.1632 | 0.5821 | |
| Unpubl. data (*Tibouchina clavata*) | Crab spider | Model | | Sit-and-wait | ... |  | Apidae | | | Hym | Social |  | -0.9216 | 0.3856 | |
| Unpubl. data (*Tibouchina clavata*) | Crab spider | Object | | Sit-and-wait | ... |  | Apidae | | | Hym | Social |  | -0.8368 | 0.3301 | |
| Unpubl. data (*Tibouchina* sp.) | Crab spider | Model | | Sit-and-wait | ... |  | Apidae | | | Hym | Social |  | -1.0888 | 0.6401 | |
| Unpubl. data (*Tibouchina* sp.) | Crab spider | Object | | Sit-and-wait | ... |  | Apidae | | | Hym | Social |  | -1.0888 | 0.7612 | |
| Unpubl. data (*Manettia luteorubra*) | Crab spider | Model | | Sit-and-wait | ... |  | Apidae | | | Hym | Social |  | -1.8563 | 0.1531 | |
| Unpubl. data (*Manettia luteorubra*) | Crab spider | Object | | Sit-and-wait | ... |  | Apidae | | | Hym | Social |  | -0.7249 | 0.1227 | |
| Unpubl. data (*Alternanthera brasiliana*) | Crab spider | Model | | Sit-and-wait | ... |  | Apidae | | | Hym | Social |  | -3.2581 | 1.0544 | |
| Unpubl. data (*Alternanthera brasiliana*) | Crab spider | Object | | Sit-and-wait | ... |  | Apidae | | | Hym | Social |  | -2.8526 | 0.5609 | |
| Unpubl. data (*Wedelia paludosa*) | Crab spider | Model | | Sit-and-wait | ... |  | Sphecidae | | | Hym | Solitary |  | 0.8473 | 0.908 | |
| Unpubl. data (*Wedelia* *paludosa*) | Crab spider | Object | | Sit-and-wait | ... |  | Sphecidae | | | Hym | Solitary |  | -0.4055 | 1.537 | |
| Unpubl. data (*Wedelia* *paludosa*) | Crab spider | Model | | Sit-and-wait | ... |  | Nymphalidae | | | Lep | ... |  | -3.3787 | 1.6713 | |
| Unpubl. data (*Wedelia* *paludosa*) | Crab spider | Object | | Sit-and-wait | ... |  | Nymphalidae | | | Lep | ... |  | -2.2801 | 0.9367 | |
| Unpubl. data (*Wedelia* *paludosa*) | Crab spider | Model | | Sit-and-wait | ... |  | ? | | | Lep | ... |  | -1.0707 | 1.1429 | |
| Unpubl. data (*Wedelia* *paludosa*) | Crab spider | Object | | Sit-and-wait | ... |  | ? | | | Lep | ... |  | -0.515 | 1.0586 | |

Notes:

^a^ Predator category: PPEv = past predation event (visual), PPEs = past predation event (scent), Live = live predators, Dead = dead predators, Model = artificial predators, Object = any abiotic structure on flowers, PR = predation risk (i.e., areas with higher predation risk, but predators were absent).

^b^ Predator native or invasive
