# Supplementary material for: A Meta-Analysis of Predation Risk Effects on Pollinator Behaviour
Source: PLoS One. 2011 Jun 13;6(6):e20689. doi: 10.1371/journal.pone.0020689 (PMC3113803; doi:10.1371/journal.pone.0020689)
Supplement: Table S2 — Sources of variation and log response ratios of predator effects on avoidance rate of pollinator behavior. (DOC) [file pone.0020689.s006.doc]

Table S2. Sources of variation and log response ratios of predator effects on avoidance rate of pollinator behaviour

|  |  |  | Predators | |  |  |  |  | Floral visitors | | |  | Log response ratio | |  |
| --- | --- | --- | --- | --- | --- | --- | --- | --- | --- | --- | --- | --- | --- | --- | --- |
| References and data source | Taxa | Category ^a^ | | Hunting Mode | Native/Invas ^b^ |  | Family | | | Order | Solit/Social |  | Effect size | Variance | |
| Unpubl. data (*Rubus rosifolius*) | Crab spider | Model | | Sit-and-wait | ... |  | Apidae | | | Hym | Social |  | 1.2947 | 0.1532 | |
| Unpubl. data (*Rubus rosifolius*) | Crab spider | Object | | Sit-and-wait | ... |  | Apidae | | | Hym | Social |  | 0.0488 | 0.2647 | |
| Unpubl. data (*Rubus rosifolius*) | Crab spider | Model | | Sit-and-wait | ... |  | Nymphalidae | | | Lep | ... |  | 1.204 | 0.127 | |
| Unpubl. data (*Rubus rosifolius*) | Crab spider | Object | | Sit-and-wait | ... |  | Nymphalidae | | | Lep | ... |  | 0.4055 | 0.1422 | |
| Unpubl. data (*Wedelia* sp. 1) | Crab spider | Model | | Sit-and-wait | ... |  | Apidae | | | Hym | Social |  | 2.3195 | 0.5876 | |
| Unpubl. data (*Wedelia* sp. 1) | Crab spider | Object | | Sit-and-wait | ... |  | Apidae | | | Hym | Social |  | 1.8436 | 0.6455 | |
| Unpubl. data (*Wedelia* sp. 1) | Crab spider | Model | | Sit-and-wait | ... |  | Vespidae | | | Hym | Social |  | 3.8712 | 1.1147 | |
| Unpubl. data (*Wedelia* sp. 1) | Crab spider | Object | | Sit-and-wait | ... |  | Vespidae | | | Hym | Social |  | 2.2336 | 1.236 | |
| Unpubl. data (*Wedelia* sp. 1) | Crab spider | Model | | Sit-and-wait | ... |  | Megachilidae | | | Hym | Solitary |  | 1.9951 | 0.5219 | |
| Unpubl. data (*Wedelia* sp. 1) | Crab spider | Object | | Sit-and-wait | ... |  | Megachilidae | | | Hym | Solitary |  | 1.0788 | 0.7086 | |
| Unpubl. data (*Wedelia* sp. 1) | Crab spider | Model | | Sit-and-wait | ... |  | Nymphalidae | | | Lep | ... |  | 1.2205 | 0.5848 | |
| Unpubl. data (*Wedelia* sp. 1) | Crab spider | Object | | Sit-and-wait | ... |  | Nymphalidae | | | Lep | ... |  | 0.9598 | 0.6207 | |
| Unpubl. data (*Wedelia* sp. 1) | Crab spider | Model | | Sit-and-wait | ... |  | Syrphidae | | | Dip | ... |  | 2.7163 | 0.5738 | |
| Unpubl. data (*Wedelia* sp. 1) | Crab spider | Object | | Sit-and-wait | ... |  | Syrphidae | | | Dip | ... |  | 2.5257 | 0.6453 | |
| Unpubl. data (*Wedelia* sp. 2) | Crab spider | Model | | Sit-and-wait | ... |  | Apidae | | | Hym | Social |  | 1.0906 | 0.2881 | |
| Unpubl. data (*Wedelia* sp. 2) | Crab spider | Object | | Sit-and-wait | ... |  | Apidae | | | Hym | Social |  | 0.5798 | 0.2594 | |
| Unpubl. data (*Wedelia* sp. 2) | Crab spider | Model | | Sit-and-wait | ... |  | Halictidae | | | Hym | Solitary |  | 2.161 | 0.6078 | |
| Unpubl. data (*Wedelia* sp. 2) | Crab spider | Object | | Sit-and-wait | ... |  | Halictidae | | | Hym | Solitary |  | 1.5433 | 0.6864 | |
| Unpubl. data (*Borreria verticillata*) | Crab spider | Model | | Sit-and-wait | ... |  | Apidae | | | Hym | Social |  | 3.862 | 1.0524 | |
| Unpubl. data (*Borreria verticillata*) | Crab spider | Object | | Sit-and-wait | ... |  | Apidae | | | Hym | Social |  | 3.4173 | 1.0747 | |
| Unpubl. data (*Borreria verticillata*) | Crab spider | Model | | Sit-and-wait | ... |  | Vespidae | | | Hym | Social |  | 2.5829 | 1.1018 | |
| Unpubl. data (*Borreria verticillata*) | Crab spider | Object | | Sit-and-wait | ... |  | Vespidae | | | Hym | Social |  | 2.0571 | 1.0496 | |
| Unpubl. data (*Borreria verticillata*) | Crab spider | Model | | Sit-and-wait | ... |  | Syrphidae | | | Dip | ... |  | 1.7395 | 0.6916 | |
| Unpubl. data (*Borreria verticillata*) | Crab spider | Object | | Sit-and-wait | ... |  | Syrphidae | | | Dip | ... |  | 1.626 | 0.746 | |
| Unpubl. data (*Tibouchina clavata*) | Crab spider | Model | | Sit-and-wait | ... |  | Apidae | | | Hym | Social |  | 1.4123 | 0.6332 | |
| Unpubl. data (*Tibouchina clavata*) | Crab spider | Object | | Sit-and-wait | ... |  | Apidae | | | Hym | Social |  | 0.1466 | 0.9743 | |
| Unpubl. data (*Tibouchina* sp.) | Crab spider | Model | | Sit-and-wait | ... |  | Apidae | | | Hym | Social |  | 0.9491 | 0.3726 | |
| Unpubl. data (*Tibouchina* sp.) | Crab spider | Object | | Sit-and-wait | ... |  | Apidae | | | Hym | Social |  | 0.8353 | 0.3641 | |
| Unpubl. data (*Manettia luteorubra*) | Crab spider | Model | | Sit-and-wait | ... |  | Apidae | | | Hym | Social |  | 1.9136 | 0.2492 | |
| Unpubl. data (*Manettia luteorubra*) | Crab spider | Object | | Sit-and-wait | ... |  | Apidae | | | Hym | Social |  | 1.135 | 0.2447 | |
| Unpubl. data (*Alternanthera brasiliana*) | Crab spider | Model | | Sit-and-wait | ... |  | Apidae | | | Hym | Social |  | 2.0541 | 0.2418 | |
| Unpubl. data (*Alternanthera brasiliana*) | Crab spider | Object | | Sit-and-wait | ... |  | Apidae | | | Hym | Social |  | 1.9169 | 0.2673 | |

Notes (Idem to Table A)
