# Supplementary material for: A Meta-Analysis of Predation Risk Effects on Pollinator Behaviour
Source: PLoS One. 2011 Jun 13;6(6):e20689. doi: 10.1371/journal.pone.0020689 (PMC3113803; doi:10.1371/journal.pone.0020689)
Supplement: Table S3 — Sources of variation and log response ratios of predator effects on pollinator behaviour (time spent on flowers). (DOC) [file pone.0020689.s007.doc]

Table S3. Sources of variation and log response ratios of predator effects on pollinator behaviour (time spent on flowers)

|  |  |  | Predators | |  |  |  |  | Floral visitors | | |  | Log response ratio | |  |
| --- | --- | --- | --- | --- | --- | --- | --- | --- | --- | --- | --- | --- | --- | --- | --- |
| References and data source | Taxa | Category ^a^ | | Hunting Mode | Native/Invas ^b^ |  | Family | | | Order | Solit/Social |  | Effect size | Variance | |
| Abbott 2006; fig.1 | ... | PPEv | | ... | ... |  | Apidae | | | Hym | Social |  | 0.5525 | 0.0833 | |
| Abbott 2006; fig.1 | ... | PPEv | | ... | ... |  | Apidae | | | Hym | Social |  | 0.3372 | 0.0661 | |
| Blancafort & Gómez 2005; table 1 | Ant | Live | | Hunter | Inv |  | Calliphoridae | | | Dip | ... |  | 0.1905 | 0.029 | |
| Blancafort & Gómez 2005; table 1 | Ant | Live | | Hunter | Inv |  | Syrphidae | | | Dip | ... |  | -0.4091 | 0.0281 | |
| Blancafort & Gómez 2005; table 1 | Ant | Live | | Hunter | Inv |  | Tachinidae | | | Dip | ... |  | 0.3864 | 0.0309 | |
| Blancafort & Gómez 2005; table 1 | Ant | Live | | Hunter | Inv |  | Sarcophagidae | | | Dip | ... |  | -0.2094 | 0.071 | |
| Elliott & Elliott 1991; table 2 | Phymatid | Live | | Sit-and-wait | Nat |  | Syrphidae | | | Dip | ... |  | -0.4406 | 0.0032 | |
| Elliott & Elliott 1991; table 2 | Phymatid | Live | | Sit-and-wait | Nat |  | Sarcophagidae | | | Dip | ... |  | 0.1148 | 0.0058 | |
| Elliott & Elliott 1991; table 2 | Phymatid | Live | | Sit-and-wait | Nat |  | Calliphoridae | | | Dip | ... |  | -0.9343 | 0.0074 | |
| Elliott & Elliott 1991; table 2 | Phymatid | Live | | Sit-and-wait | Nat |  | Muscidae | | | Dip | ... |  | -0.0619 | 0.025 | |
| Elliott & Elliott 1991; table 2 | Phymatid | Live | | Sit-and-wait | Nat |  | Chalcididae | | | Hym | Solitary |  | 0.3106 | 0.0065 | |
| Elliott & Elliott 1991; table 2 | Phymatid | Live | | Sit-and-wait | Nat |  | Vespidae | | | Hym | Social |  | -3.6045 | 0.0142 | |
| Elliott & Elliott 1991; table 2 | Phymatid | Live | | Sit-and-wait | Nat |  | Sphecidae | | | Hym | Solitary |  | -1.3766 | 0.0273 | |
| Elliott & Elliott 1991; table 2 | Phymatid | Live | | Sit-and-wait | Nat |  | Halictidae | | | Hym | Solitary |  | -1.6953 | 0.0183 | |
| Elliott & Elliott 1991; table 2 | Phymatid | Live | | Sit-and-wait | Nat |  | Chrysomelidae | | | Col | ... |  | -0.945 | 0.0229 | |
| Elliott & Elliott 1991; table 2 | Phymatid | Live | | Sit-and-wait | Nat |  | Cantharidae | | | Col | ... |  | 0.2533 | 0.0183 | |
| Elliott & Elliott 1994; table 1 | Phymatid | Live | | Sit-and-wait | Nat |  | Several | | | Several | ... |  | -0.7638 | 0.0488 | |
| Elliott & Elliott 1994; table 2 | Phymatid | Live | | Sit-and-wait | Nat |  | Apidae | | | Hym | Social |  | -0.7191 | 0.0735 | |
| Elliott & Elliott 1994; table 3 | Phymatid | Live | | Sit-and-wait | Nat |  | Vespidae | | | Hym | Social |  | -0.6702 | 0.109 | |
| Hansen & Müller 2009; fig. 2c | Ant | Live | | Hunter | Inv |  | Gekkonidae | | | Squamata | ... |  | -2.3638 | 0.2219 | |
| Hansen & Müller 2009; fig. 2c | Ant | Live | | Hunter | Inv |  | Gekkonidae | | | Squamata | ... |  | -2.6522 | 0.1727 | |
| Junker et al. 2007; text | Ant | Live | | Hunter | Nat |  | Apidae | | | Hym | Social |  | -0.7259 | 0.0344 | |
| Junker et al. 2007; text | Ant | Live | | Hunter | Nat |  | Apidae | | | Hym | Social |  | -1.6645 | 0.3019 | |
| Lach 2008a; text | Ant | Live | | Hunter | Inv |  | Apidae | | | Hym | Social |  | -1.3157 | 0.0171 | |
| Lach 2008b; table 3 | Ant | Live | | Hunter | Inv |  | Colletidae | | | Hym | Solitary |  | -0.2199 | 0.4791 | |
| Lach 2008b; table 3 | Ant | Live | | Hunter | Inv |  | Apidae | | | Hym | Social |  | -0.0855 | 0.4744 | |
| Lach 2008b; table 3 | Ant | Live | | Hunter | Inv |  | Apidae | | | Hym | Social |  | 0.1066 | 0.7603 | |
| Lima 1991; fig. 2a | ... | PR | | ... | ... |  | Trochilidae | | | Trochiliformes | ... |  | -0.1483 | 0.0159 | |
| Munõz & Arroyo 2004; fig. 2 | Bird | Live | | Hunter | Nat |  | Satiridae | | | Lep | ... |  | -0.2571 | 0.226 | |
| Munõz & Arroyo 2004; fig. 2 | Lizard | Live | | Sit-and-wait | Nat |  | Satiridae | | | Lep | ... |  | -0.5285 | 0.2477 | |
| Munõz & Arroyo 2004; fig. 2 | Bird | Live | | Hunter | Nat |  | Syrphidae | | | Dip | ... |  | 0.2285 | 0.1673 | |
| Munõz & Arroyo 2004; fig. 2 | Lizard | Live | | Sit-and-wait | Nat |  | Syrphidae | | | Dip | ... |  | -1.3879 | 0.4661 | |
| Munõz & Arroyo 2004; fig. 2 | Bird | Live | | Hunter | Nat |  | Andrenidae | | | Hym | Solitary |  | -0.0603 | 0.4278 | |
| Munõz & Arroyo 2004; fig. 2 | Lizard | Live | | Sit-and-wait | Nat |  | Andrenidae | | | Hym | Solitary |  | -0.0603 | 0.4078 | |
| Ness 2006; table 1 | Ant | Live | | Hunter | Nat |  | Several | | | Hym | ? |  | -1.0531 | 0.1179 | |
| Ness 2006; table 1 | Ant | Live | | Hunter | Nat |  | Several | | | Hym | ? |  | 0.5695 | 0.1404 | |
| Suttle 2003; fig 2, text | Crab spider | Live | | Sit-and-wait | Nat |  | Several | | | Several | ? |  | -1.2528 | 0.1041 | |

Notes (idem to Table A)
