# Supplementary material for: A Meta-Analysis of Predation Risk Effects on Pollinator Behaviour
Source: PLoS One. 2011 Jun 13;6(6):e20689. doi: 10.1371/journal.pone.0020689 (PMC3113803; doi:10.1371/journal.pone.0020689)
Supplement: Table S4 — Sources of variation and log response ratios of predator effects on visitation rate of pollinators of different biomass. (DOC) [file pone.0020689.s008.doc]

Table S4. Sources of variation and log response ratios of predator effects on visitation rate of pollinators of different biomass

|  |  |  | Predator | |  |  | Floral visitors | | | | |  |  | Log response ratio | |
| --- | --- | --- | --- | --- | --- | --- | --- | --- | --- | --- | --- | --- | --- | --- | --- |
| References and data source | Taxa | | | Category ^a^ | |  | Species/Morfosp. | Order | Fresh biomass | Dry biomass | Size class ^b^ | |  | Effect size | Variance |
| Brechbühl et al. 2010a, fig. 1 | Crab spider | | | Live | |  | *Colletes* sp. | Hym |  | 15.6 | medium | |  | -0.147 | 0.1686 |
| Brechbühl et al. 2010a, fig. 1 | Crab spider | | | Live | |  | *Halictus* sp. | Hym |  | 6.2 | small | |  | -0.0812 | 0.1585 |
| Brechbühl et al. 2010a, fig. 1 | Crab spider | | | Live | |  | *Hylaeus* sp. | Hym |  | 5.3 | small | |  | -0.3677 | 0.0798 |
| Brechbühl et al. 2010a, fig. 1 | Crab spider | | | Live | |  | *Lasioglossum* sp. | Hym |  | 5.2 | small | |  | -0.2877 | 0.1026 |
| Brechbühl et al. 2010a, fig. 1 | Crab spider | | | Live | |  | *Bombus* spp. | Hym |  | 97.8 | large | |  | -0.0741 | 0.221 |
| Brechbühl et al. 2010a, fig. 1 | Crab spider | | | Live | |  | *Apis mellifera* | Hym |  | 29.5 | large | |  | -0.0741 | 0.1782 |
| Brechbühl et al. 2010a, fig. 1 | Crab spider | | | Live | |  | *Syritta* sp. | Dip |  | 1.3 | small | |  | -0.0953 | 0.2025 |
| Brechbühl et al. 2010a, fig. 1 | Crab spider | | | Live | |  | *Sphaerophoria* sp. | Dip |  | 2.6 | small | |  | -0.1222 | 0.2153 |
| Brechbühl et al. 2010a, fig. 1 | Crab spider | | | Live | |  | *Eristalis tenax* | Dip |  | 17.2 | medium | |  | -0.0728 | 0.1969 |
| Dukas & Morse 2003, fig. 2 | Crab spider | | | Live | |  | *Apis mellifera* | Hym | 85.4 |  | medium | |  | -0.1865 | 0.1774 |
| Dukas & Morse 2003, fig. 2 | Crab spider | | | Live | |  | *Bombus ternarius* | Hym | 81.55 |  | medium | |  | -0.1082 | 0.1071 |
| Dukas & Morse 2003, fig. 2 | Crab spider | | | Live | |  | *Bombus vagans* | Hym | 109.39 |  | large | |  | -0.018 | 0.0848 |
| Dukas & Morse 2003, fig. 2 | Crab spider | | | Live | |  | *Bombus terricola* | Hym | 184.28 |  | large | |  | -0.0853 | 0.3236 |
| Dukas & Morse 2005, fig. 2 | Crab spider | | | Live | |  | *Apis mellifera* | Hym | 85.4 |  | medium | |  | -1.5531 | 2.2937 |
| Dukas & Morse 2005, fig. 2 | Crab spider | | | Live | |  | *Bombus ternarius* | Hym | 81.55 |  | medium | |  | 0 | 0.4147 |
| Dukas & Morse 2005, fig. 2 | Crab spider | | | Live | |  | *Bombus vagans* | Hym | 109.39 |  | large | |  | 0.1633 | 0.5177 |
| Unpubl. data (*Rubus rosifolius*) | Crab spider | | | Model | |  | *Augochlora* sp. | Hym | 21 |  | small | |  | -2.3026 | 1.5329 |
| Unpubl. data (*Rubus rosifolius*) | Crab spider | | | Model | |  | *Stilbochlora* sp. | Hym | 20 |  | small | |  | -1.0986 | 1.49 |
| Unpubl. data (*Rubus rosifolius*) | Crab spider | | | Model | |  | *Trigona* sp. | Hym | 33 |  | small | |  | -1.7525 | 0.1762 |
| Unpubl. data (*Rubus rosifolius*) | Crab spider | | | Model | |  | *Aeria olena* | Lep | 20 |  | small | |  | -3.091 | 1.2025 |
| Unpubl. data (*Wedelia* sp. 1) | Crab spider | | | Model | |  | *Trigona* sp. | Hym | 48.5 |  | small | |  | -1.2973 | 0.6205 |
| Unpubl. data (*Wedelia* sp. 1) | Crab spider | | | Model | |  | *Apis mellifera* | Hym | 86.5 |  | medium | |  | -1.1394 | 1.6076 |
| Unpubl. data (*Wedelia* sp. 1) | Crab spider | | | Model | |  | *Agelaia vicina* | Hym | 30.5 |  | small | |  | -2.5829 | 0.63 |
| Unpubl. data (*Wedelia* sp. 1) | Crab spider | | | Model | |  | Megachilidae sp. | Hym | 84.2 |  | medium | |  | -0.8883 | 0.4055 |
| Unpubl. data (*Wedelia* sp. 2) | Crab spider | | | Model | |  | *Apis mellifera* | Hym | 93.2 |  | medium | |  | -2.4889 | 1.1237 |
| Unpubl. data (*Wedelia* sp. 2) | Crab spider | | | Model | |  | Halictinae sp. | Hym | 10.7 |  | small | |  | -2.9463 | 1.0622 |
| Unpubl. data (*Borreria verticillata*) | Crab spider | | | Model | |  | *Apis mellifera* | Hym | 86.5 |  | medium | |  | -3.2581 | 0.3432 |
| Unpubl. data (*Borreria verticillata*) | Crab spider | | | Model | |  | Vespidae sp. | Hym | 52.7 |  | medium | |  | -1.1026 | 1.9576 |
| Unpubl. data (*Tibouchina clavata*) | Crab spider | | | Model | |  | *Trigona* sp. | Hym | 22.7 |  | small | |  | -0.9266 | 0.4006 |
| Unpubl. data (*Tibouchina clavata*) | Crab spider | | | Model | |  | *Bombus* sp. 1 | Hym | 346.2 |  | large | |  | -0.922 | 0.4039 |
| Unpubl. data (*Tibouchina clavata*) | Crab spider | | | Model | |  | *Bombus* sp. 2 | Hym | 624.4 |  | large | |  | -0.4806 | 1.8587 |
| Unpubl. data (*Tibouchina clavata*) | Crab spider | | | Model | |  | *Xylocopa* sp. | Hym | 863 |  | large | |  | -0.8514 | 0.2524 |
| Unpubl. data (*Tibouchina clavata*) | Crab spider | | | Model | |  | Halictinae sp. | Hym | 27.2 |  | small | |  | -0.2412 | 0.6979 |
| Unpubl. data (*Tibouchina* sp.) | Crab spider | | | Model | |  | *Trigona* sp. | Hym | 19.8 |  | small | |  | -1.0888 | 0.6401 |
| Unpubl. data (*Manettia luteorubra*) | Crab spider | | | Model | |  | *Trigona* sp. | Hym | 19.8 |  | small | |  | -1.8563 | 0.1531 |
| Unpubl. data (*Alternanthera brasiliana*) | Crab spider | | | Model | |  | *Apis mellifera* | Hym | 93.3 |  | medium | |  | -3.2581 | 1.0544 |

Notes:

^a^ Predator category: Idem Table A.

^b^ Pollinator size: Small = up to 50 g, Medium = 50.01 to 100 g, Large = higher than 100 g.
